# Endothelial repair in stented arteries is accelerated by inhibition of Rho-associated protein kinase

**DOI:** 10.1093/cvr/cvw210

**Published:** 2016-09-26

**Authors:** Sarah T. Hsiao, Tim Spencer, Luke Boldock, Svenja Dannewitz Prosseda, Ioannis Xanthis, Francesco J. Tovar-Lopez, Heleen M. M. Van Beusekom, Ramzi Y Khamis, Nicolas Foin, Neil Bowden, Adil Hussain, Alex Rothman, Victoria Ridger, Ian Halliday, Cecile Perrault, Julian Gunn, Paul C. Evans

**Affiliations:** 1 Department of Infection, Immunity and Cardiovascular Disease, University of Sheffield, Sheffield S10 2RX, UK; 2 INSIGNEO Institute of In Silico Medicine, University of Sheffield, Sheffield S10 2RX, UK; 3 Materials and Engineering Research Institute, Sheffield Hallam University, Sheffield S1 4RF, UK; 4 Department of Mechanical Engineering, University of Sheffield, Sheffield S10 2RX, UK; 5 School of Electrical and Computer Engineering, RMIT University, Melbourne VIC 3001, Australia; 6 Department of Cardiology, ERASMUS MC, Rotterdam 3015 CE, the Netherlands; 7 Faculty of Medicine, National Heart and Lung Institute, Imperial College London WI2 0HS, UK; 8 National Heart Centre, Singapore 169609; 9 Bateson Centre, University of Sheffield, Sheffield S10 2RX, UK

**Keywords:** Endothelial cells, Stent, Shear stress, ROCK, Fasudil

## Abstract

**Aims:**

Stent deployment causes endothelial cells (EC) denudation, which promotes in-stent restenosis and thrombosis. Thus endothelial regrowth in stented arteries is an important therapeutic goal. Stent struts modify local hemodynamics, however the effects of flow perturbation on EC injury and repair are incompletely understood. By studying the effects of stent struts on flow and EC migration, we identified an intervention that promotes endothelial repair in stented arteries.

**Methods and Results:**

*In vitro* and *in vivo* models were developed to monitor endothelialization under flow and the influence of stent struts. A 2D parallel-plate flow chamber with 100 μm ridges arranged perpendicular to the flow was used. Live cell imaging coupled to computational fluid dynamic simulations revealed that EC migrate in the direction of flow upstream from the ridges but subsequently accumulate downstream from ridges at sites of bidirectional flow. The mechanism of EC trapping by bidirectional flow involved reduced migratory polarity associated with altered actin dynamics. Inhibition of Rho-associated protein kinase (ROCK) enhanced endothelialization of ridged surfaces by promoting migratory polarity under bidirectional flow (*P* < 0.01). To more closely mimic the *in vivo* situation, we cultured EC on the inner surface of polydimethylsiloxane tubing containing Coroflex Blue stents (65 μm struts) and monitored migration. ROCK inhibition significantly enhanced EC accumulation downstream from struts under flow (*P* < 0.05). We investigated the effects of ROCK inhibition on re-endothelialization *in vivo* using a porcine model of EC denudation and stent placement. *En face* staining and confocal microscopy revealed that inhibition of ROCK using fasudil (30 mg/day via osmotic minipump) significantly increased re-endothelialization of stented carotid arteries (*P* < 0.05).

**Conclusions:**

Stent struts delay endothelial repair by generating localized bidirectional flow which traps migrating EC. ROCK inhibitors accelerate endothelial repair of stented arteries by enhancing EC polarity and migration through regions of bidirectional flow.

## 1. Introduction

Interventions used routinely to treat arterial disease, including balloon angioplasty and stent implantation, lead to damage and loss of endothelial cells (EC). Given the essential role of EC in suppressing inflammation and thrombosis, the restoration of functional vascular endothelium is an important therapeutic goal to avoid the lethal consequences of in-stent restenosis and thrombosis.^1^^,^^2^ There are several factors that can potentially influence re-endothelialization in stented arteries, one of which is the presence of the stent itself that provides a non-physiological surface for cell adhesion and alters blood flow.^3–5^ However, the effect of stent deployment on EC repair remains poorly understood. This issue is particularly relevant in the era of drug-eluting stents, which release cytostatic compounds to prevent restenosis. They are coated with sirolimus (Cypher) that targets the mammalian target of rapamycin pathway or paclitaxel (Taxus) that targets microtubules to suppress cell motility and mitosis. Although drug-eluting stents have effectively reduced rates of restenosis, they have also been associated with a higher incidence of late and very late thrombosis.^1^^,^^2^^,^^6^ These deleterious effects have been attributed, in part, to the effects of cytostatic compounds on vascular endothelium leading to delayed healing and subsequent exposure of stent struts for thrombus initiation.^6–8^ There is therefore a need to develop new strategies to accelerate endothelialization of stented arteries and thereby reduce the incidence of late and very late thrombosis.

The repair of injured vascular endothelium involves migration of EC from adjacent uninjured sites.^8^^,^^9^ Cell migration is a tightly co-ordinated process that requires the establishment of front–rear polarity. It involves the formation of a membrane protrusion at the cell front via the polymerization of actin filaments.^10^ The subsequent development of adhesions links the actin cytoskeleton to the extracellular matrix, thus allowing the generation of traction forces via actomyosin contraction. On the other hand, sustained migration requires the severing of actin filaments at the rear of the cell for subsequent rounds of actin nucleation. Rho-associated protein kinase (ROCK) controls the migration cascade by promoting phosphorylation of actin-binding proteins including myosin light chain (MLC) and cofilin which control actin dynamics, actomyosin contractility and cell polarity.^11–13^

The deployment of a stent by balloon angioplasty leads to major changes in vascular mechanics. Of note, the presence of stent struts alters local blood flow patterns thereby modifying wall shear stress (WSS),^3–5^ a frictional force that is exerted by flowing blood on the vessel wall. Alterations in WSS induced by stent application have been correlated with neointimal hyperplasia^4^ and we hypothesize that they may also influence the physiology of EC, which are exquisitely sensitive to flow.

Here we used *in vitro* and *in vivo* models to study the influence of stent struts on local hemodynamics and EC migration. Stent struts generated disturbed flow patterns which reduced EC polarization and impeded migration towards cell-free space. Inhibition of ROCK enhanced EC migration over stent struts by promoting migratory polarization of cells via modulating the activity of MLC and cofilin. We conclude that ROCK inhibitors may have beneficial effects in stented arteries by promoting re-endothelialization and thus restoring vascular homeostasis.

## 2. Methods

### 2.1 Study approval

For studies of human cells, experiments were approved by University of Sheffield Research Ethics Committee (reference 10/H1308/25) and all subjects gave informed consent. Studies using human cells were used in accordance to the standards set by the Declaration of Helsinki. For animal studies, all procedures were approved by the University of Sheffield ethics committee and performed in accordance with the UK Home Office Animals (Scientific Procedures) Act 1986 and in accordance with Directive 2010/63/EU of the European Parliament on the protection of animals used for scientific purposes.

### 2.2 EC culture and application of flow

Pharmacological inhibition of ROCK activity was performed using Y27632 (Calbiochem) or fasudil (5-(1,4-Diazepane-1-sulfonyl) isoquinoline; HA-1077; Calbiochem) at 2 µM. Silencing of ROCK1 and ROCK2 was performed using small interfering RNA (siRNA; ON-TARGETplus Human ROCK1 siRNA SMARTpool and ON-TARGETplus Human ROCK2 siRNA SMARTpool). Human umbilical vein EC (HUVEC) were isolated using collagenase digestion. Human coronary artery EC (HCAEC) were obtained commercially (PromoCell, Heidelberg, Germany). EC were seeded into polydimethylsiloxane (PDMS) flow chambers with ridges (100 µm high, 100 µm length) or onto flat flow chambers (Ibidi fibronectin-coated µ-Slide I^0.6^, Ibidi GmbH). Flowing medium was applied using the Ibidi pump system and chamber slides were placed on the stage of an inverted light microscope (Nikon® TE300) enclosed in a Perspex box warmed to 37 °C. Time-lapse imaging was performed for up to 96 h. Individual cells were manually tracked using ImageJ software. 3D stented model vessels were fabricated with PDMS and the internal surface was coated with fibronectin prior to deployment of a Coroflex Blue stent. EC were seeded as a confluent monolayer upstream of the first stent strut. Flowing medium was applied using the Ibidi® pump system in a 5% CO_2_ humidified atmosphere at 37 °C. EC were identified by light microscopy. Multiple independent experiments were conducted using primary cells isolated from different individuals, and the number of independent experiments carried out is stated in the figure legends.

### 2.3 Immunofluorescent staining of cultured EC

Cell polarity was assessed by immunofluorescent staining using antibodies against β-tubulin (Cell Signalling Technology) and Alexafluor568-conjugated secondary antibodies (Invitrogen) to identify the microtubular organizing centre (MTOC), phalloidin-488 (Cell Signalling Technology) to identify actin. Nuclei were identified using DAPI (Sigma). Imaging was carried out using an inverted fluorescence microscope (Olympus IX71) and image analysis was performed using Image J software (1.49p). Polarized cells were defined as those with an elongated morphology with the MTOC positioned upstream from the nucleus as described.^13^

### 2.4 Western blotting and enzyme-linked immunosorbent assay

Total cell lysates were isolated using lysis buffer (Tris 25 mM, sodium chloride 150 mM, 0.1% Sodium dodecyl sulphate, 0.5% sodium deoxycholate, 1% Triton X100). Western blotting was carried out using specific antibodies against phosphorylated cofilin (Cell Signalling Technology), phosphorylated MLC (Cell Signalling Technology), and pyruvate dehydrogenase complex component X (PDHX; Cell Signalling Technology) with horse radish peroxidase-conjugated secondary antibodies (Dako). Chemiluminescent detection was carried out using ECL Prime^®^ (GE Healthcare). The protein levels of tissue factor pathway inhibitor (TFPI) or von Willebrand factor (vWF) in cell lysates or cell culture supernatants were quantified by enzyme-linked immunosorbent assay (ELISA; AbCam).

### 2.5. *In vivo* porcine stent model

Male Yorkshire White pigs (22–27 kg) were studied. EC denudation of carotid arteries was achieved by inflating a balloon 5 times for 30 s at a balloon/artery diameter ratio of 1.2:1. A Coroflex™ stent was deployed over the denuded region. Animals were then treated with fasudil (30 mg/day) or with vehicle alone using osmotic minipumps. Aspirin (150 mg/day) and clopidogrel (75 mg/day) were given orally for 3 days following stent implantation. After 3 days, EC coverage of the stented segment was determined by *en face* staining using anti-PECAM-1 antibodies followed by confocal microscopy.

### 2.6 Computational fluid dynamics

Fluid flow in the 2D (3D) chamber was studied using the two-dimensional [D2Q9 (D3Q19)] two-relaxation-time (TRT) incompressible lattice Boltzmann (LB) method^14^^,^^15^ to solve the isothermal incompressible Navier-Stokes and Continuity equations. A TRT magic parameter^15^ of 3/16 was used and the inlet (outlet) parabolic-velocity (constant-pressure) was fixed whilst the inlet (outlet) pressure (velocity) were linearly extrapolated. LB node spacing was 10/3 μm with an upstream (downstream) free region of 2 mm (2.6 mm) to avoid entrance (exit) disturbances. WSS values were obtained from quadratic extrapolations from near wall sites. The fluid was taken as Newtonian water (shear viscosity, *η* = 10^−^^3^ Pa.s, density, *ρ* = 10^3^ kg/m^3^) and steady flow simulations were carried out at Reynolds numbers, $Re=\frac{U^{max}H\rho }{\eta }=117$ corresponding to an open channel WSS values of 13 dyn/cm^2^ respectively (*H* = 600μm, *U*^max^ = maximum inlet velocity). Studies of fluid flow in stented PDMS tubes or arteries required import of cross-sectional lumen slices from μCT analysis to CFD analysis using the marker-cell method with matched voxel resolution ($Re=\;\overset{-}{U}D_{h}\rho /\eta$ where $\overset{-}{U}$= mean velocity and $D_{h}$ = mean diameter*)*. For stented PDMS tubes, WSS measurements were extrapolated as for the 2D system and taken from steady state flow *Re* = 410 with the Newtonian viscosity taken as that of culture medium (*η*= 7.3×10^−4^ Pa.s, *ρ* = 1005 kg/m^3^). For stented carotid arteries, *Re *= 150 with the Newtonian viscosity taken as blood (*η*= 3.7×10^−^^3^ Pa.s, *ρ* = 1060 kg/m^3^).

### 2.7 Statistical analysis

All experiments were performed at least three times and data are presented as mean ± standard error mean. Statistical significance was determined using either Student’s *t-*test or *one-way* ANOVA followed by a *Bonferroni* multiple comparison *post hoc* test when appropriate using the Prism6 software. A *P* value of less than 0.05 was considered to be statistically significant.

## 3. Results

### 3.1 A 2D *in vitro* model of EC migration over stent strut geometries

**Figure 1:**
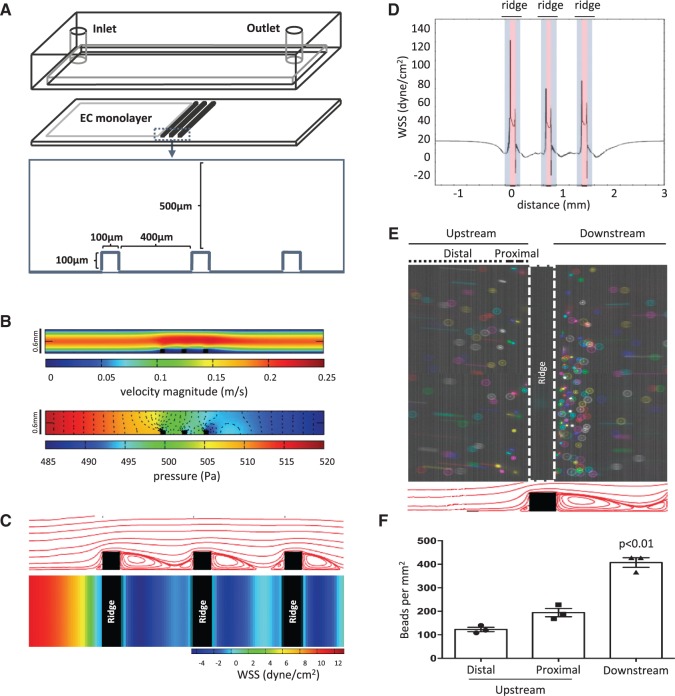
Fluid flow in the ridged chamber slide. (*A*) Configuration of the ridged flow chamber. (*B–D*) CFD were carried out to simulate flow patterns and WSS magnitude assuming an inlet flow rate of 21.6 ml/min. Flow is from left to right. (*B*) The average velocity (upper) and pressure gradient (lower) along the chamber slide are shown. (*C*) The flow path (upper, cross-sectional) and time-averaged WSS map (lower, upper surface view) are shown. (*D*) The unfolded WSS map along the surface of the ridges. Grey-shaded area represents the vertical planes of the ridge and the red-shaded area represents the horizontal plane of the ridge. (*E* and *F*) The ridged slide was exposed to flowing water and particle imaging velocimetry was performed. (*E*) Collated time-lapse images of fluorescently labelled microspheres are shown. A total of seven images were merged and each image was represented with a different colour (upper panel, upper surface view). Flow patterns predicted from CFD are shown for reference (lower panel, cross-sectional). (*F*) Microspheres accumulating at the upstream distal, upstream proximal, and downstream regions were quantified. Data were pooled from three independent experiments and mean values +/− SEM are shown alongside individual data points. Differences between means were compared using a one-way ANOVA.

To study the influence of stent strut geometries on flow patterns and consequent effects on EC migration, we developed an *in vitro* flow chamber that contained three ridges that were 100 µm high and 100 µm wide and separated by 400 µm (approximating the geometry of second generation stent struts). The chamber was linked to a continuous-pump flow system with ridges positioned perpendicular to the flow direction (*Figure 1A*) and coupled to a live cell imaging platform. CFD demonstrated that cell culture medium introduced at a flow rate of 21.6 ml/min would generate flow velocities that were highest through the centre (0.25 m/s) and decreased in magnitude when approaching the top and bottom of the chamber (*Figure 1B top*). The pressure along the length of the parallel plates was mostly homogeneous with a gradual 5% decrease, while the presence of ridges generated variation in the pressure gradient around these features (*Figure 1B* bottom). Mapping the WSS revealed that it was relatively high (13 dyn/cm^2^) and unidirectional at the inlet, whereas areas immediately upstream and downstream from the ridges were exposed to a low time-averaged WSS values, correlating with recirculating flow patterns observed in the flow path analysis (*Figure 1C*). In detail, WSS was reduced to 0 dyn/cm^2^ just upstream of the ridge, followed by a sharp increase in WSS along the first vertical plane of the ridge (blue) that peaked at > 100 dyn/cm^2^ at the interface between vertical and horizontal planes of the ridge (*Figure 1D*). The WSS remained elevated (30 dyn/cm^2^) on top of the ridge (red), decreased sharply along the second vertical plane of the ridge (blue) and remained low in the flow recirculation zone downstream from the ridge. The pattern of WSS variation repeated for each of the ridges. The disruption in flow was confirmed by particle imaging velocimetry which revealed that the accumulation of polystyrene beads was greater downstream from the ridge (407 ± 35 beads per mm^2^) where a large recirculating zone was present, compared to a site immediately upstream (proximal; 195 ± 30 beads per mm^2^) or further upstream (distal; 123 ± 16 beads per mm^2^) from the ridge (*Figure 1E* and *F*). Overall, these data indicate that the ridges alter high unidirectional flow (at the inlet) to create local flow disturbances including a region of very high unidirectional WSS at the top of the ridge and recirculating bidirectional flow with low-average WSS downstream from the ridge (herein referred to as bidirectional flow).

### 3.2 EC migration was impeded at sites of bidirectional flow located downstream from ridges

**Figure 2:**
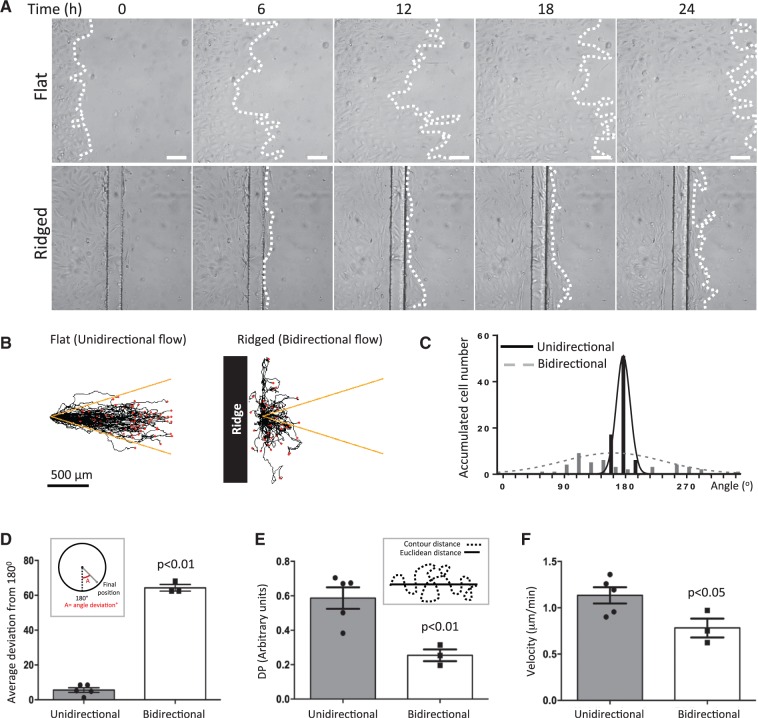
The migration pattern of EC was disrupted under bidirectional flow. HUVEC were seeded on flat or ridged slides (upstream from the first ridge) and their migration under flow was monitored by time-lapse imaging for 72 h. (*A*) Representative images. Flow at inlet is from left to right. White dotted lines represent leading edge of monolayers. White bar = 100 μm. (*B–D*) Single cell tracking analysis of cells exposed to unidirectional or bidirectional (downstream from ridge) flow. (*B*) Migration paths are shown. Flow at inlet is from left to right. Each red dot represents a cell. Yellow lines indicate a 15% deviation from the inlet flow direction. (*C* and *D*) For each cell, the angle between its final position and the inlet flow direction (180°) was calculated (angle deviation). (*C*) Angle deviations were divided into 20 × 18° groups and the number of cells in each group is shown. (*D*) Angle deviations, (*E*) directional persistence and (*F*) average velocities were calculated. Data were pooled from five independent experiments and mean values +/− SEM are shown alongside individual data points. Differences between means were compared using an unpaired *t*-test.

To determine the effect of ridges on EC migration, HUVEC were seeded onto the ridged flow chamber (between the inlet and the first ridge) or onto a flat surface as a control. Flow was applied to the chamber and EC migration monitored by live cell imaging. Cells migrated over the flat surface in the direction of flow and covered a distance of > 500 µm in 24 h (*Figure 2A top* and Supplementary material online, *Movie 1*). Cells on the ridged slide negotiated the first ridge (despite the elevated WSS at the upper surface) and reached the region downstream of the ridge effectively (*Figure 2A bottom* and Supplementary material online, *Movie 2*). However, EC did not migrate beyond the region of bidirectional flow located downstream from the ridges (*Figure 2A* compare *top* with *bottom* and Supplementary material online, *Movie 2*).

These features were assessed by single cell tracking which revealed unified forward migration in the flow direction in cells exposed to unidirectional flow (*Figure 2B left*), but less uniform direction in cells under bidirectional flow (*Figure 2B right*). This was quantified by measuring the angle between the radial axis (joining the origin and final position of the cell) and the flow axis (nominated 0°), which was wider in cells exposed to bidirectional compared to unidirectional flow (*Figure 2C* and *D*). The directional persistence, which is a ratio of the Euclidean distance: accumulated contour distance travelled and therefore a measure of the uniformity of migration direction, was also significantly reduced in cells exposed to bidirectional compared to unidirectional flow (*Figure 2E*). In addition to the loss of directionality in HUVEC under bidirectional flow, the average velocity (0.78 ± 0.10 µm/min) was also significantly reduced compared to cells under unidirectional flow (1.13 ± 0.09 µm/min; *Figure 2F*). These data indicate that the direction and velocity of HUVEC migration were impeded downstream from the ridge. Thus we propose that EC in the upstream region move into free space effectively because their migration is promoted by unidirectional flow, whereas EC in the downstream region become ‘trapped’ because bidirectional flow at that site exerts opposing directional cues.

### 3.3 ROCK inhibitors enhance EC migration under bidirectional flow

**Figure 3:**
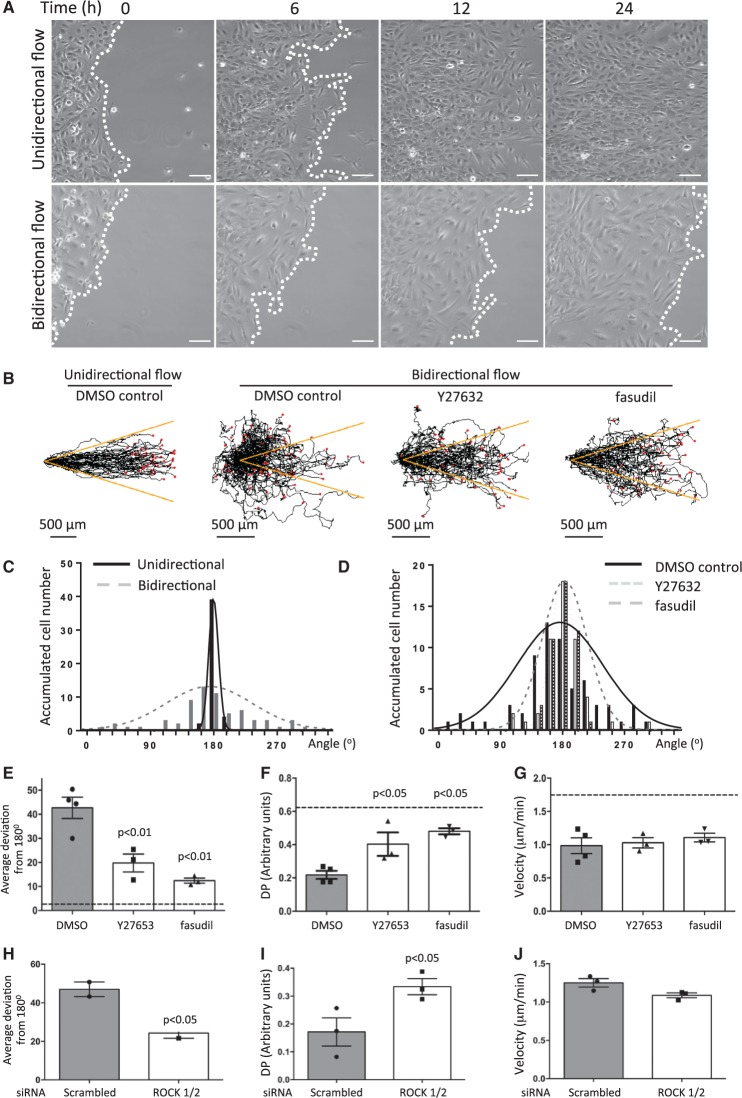
ROCK inhibition enhanced EC migration into cell-free space under bidirectional flow. (*A*) HUVEC were seeded onto Ibidi slides, exposed to unidirectional or bidirectional flow and migration was monitored. Representative images are shown. Flow at inlet is from left to right. White dotted lines represent leading edge of monolayers. White bar = 100 μm. (*B–G*) HUVEC were seeded onto Ibidi slides and exposed to unidirectional or bidirectional flow in the presence of a ROCK inhibitor (2 μM Y27632 or 2 μM fasudil) or DMSO vehicle and migration was monitored. (*B*) Migration paths. Each red dot represents a cell. Yellow lines indicate a 15% deviation from the inlet flow direction. (*C–E*) For each cell, the angle between its final position and the inlet flow direction (180°) was calculated (angle deviation). (*C* and *D*) Angle deviations were divided into 20 × 18° groups and the number of cells in each group is shown. (*E*) Angle deviations, (*F*) directional persistence (DP; contour distance/Euclidean distance) (*G*) and migration velocities were calculated. Data were pooled from four independent experiments and mean values +/− SEM are shown alongside individual data points. (*H–J*) EC were treated with siRNA against ROCK1 and ROCK2 (siROCK1/2) or with non-targeting scrambled sequences, exposed to unidirectional or bidirectional flow and migration was monitored. (*H*) Angle deviations, (*I*) directional persistence (DP), and (*J*) average velocities were calculated. Data were pooled from three independent experiments and mean values +/− SEM are shown alongside individual data points. Differences between means were compared using a one-way ANOVA (*E–G*) or unpaired *t*-test (*H–J*).

We hypothesized that interventions that uncouple EC from flow-mediated directional cues may allow EC to migrate from regions of bidirectional flow into cell free space. This was tested using inhibitors of ROCK, a molecule that controls EC polarization in response to flow.^10–12^^,^^16^^,^^17^ Studies were carried out using HUVEC or HCAEC cultured on commercial slides exposed to bidirectional flow (± 4 dyn/cm^2^ at 0.1 Hz) or unidirectional flow (13 dyn/cm^2^) as a control. It was observed that HUVEC exposed to bidirectional flow exhibited delayed migration into cell-free space (*Figure 3A* compare *top* and *bottom* panels, and compare Supplementary material online, *Movies 3 and 4*), a greater deviation in migration from the flow axis (*Figure 3B, C,* and Supplementary material online, *Figure S1A* and *B*) and reduced velocity (Supplementary material online, *Figure S1C*) compared to cells exposed to unidirectional flow. Treatment with pharmacological ROCK inhibitors (Y27632 or fasudil) promoted migration along the flow axis (*Figure 3B, D*, and *E*), and significantly enhanced directional persistence (*Figure 3F*) in cells exposed to bidirectional flow whereas there was no effect on migration velocity (*Figure 3G*). Similar observations were made using HCAEC which exhibited a greater deviation in migration from the flow axis, reduced directional persistence and reduced velocity compared to cells exposed to unidirectional flow (Supplementary material online, *Figure S2A–D*). Inhibition of ROCK enhanced migration along the flow axis and increased directional persistence without altering migration velocity in HCAEC exposed to bidirectional flow (Supplementary material online, *Figure S2E* and *G*). Similar effects were also generated by siRNA-mediated silencing of ROCK1/2 (Supplementary material online, *Figure S3*) which promoted HUVEC migration along the flow axis (*Figure 3H*) and enhanced directional persistence (*Figure 3I*) in cells exposed to bidirectional flow without altering average velocity (*Figure 3J*). Thus inhibition of ROCK promoted EC migration into cell-free space under bidirectional flow.

**Figure 4:**
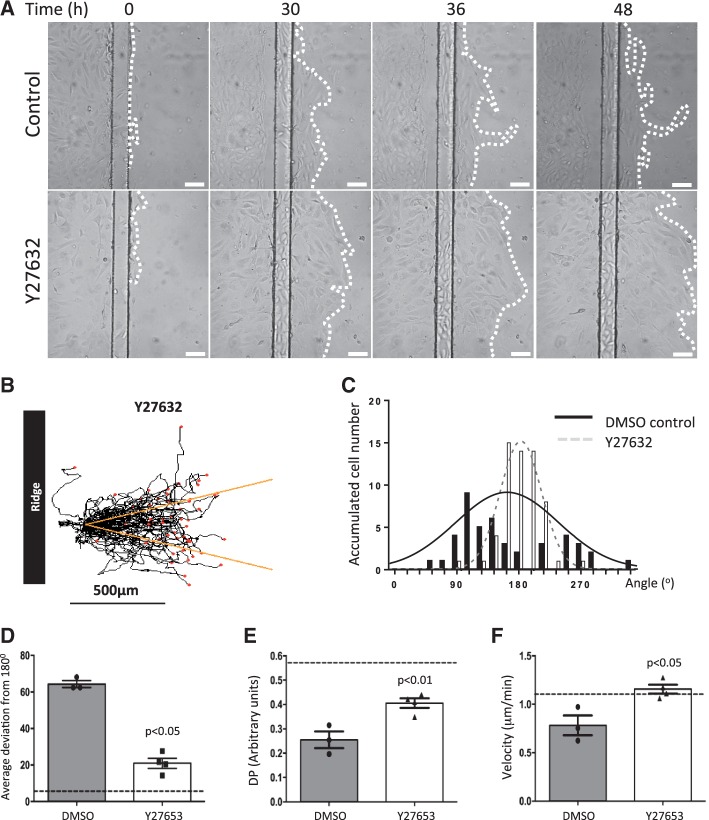
ROCK inhibition promoted EC migration on a ridged surface exposed to bidirectional flow. (*A*) HUVEC were seeded onto ridged slides upstream from the first ridge, exposed to flow in the presence of a ROCK inhibitor (2 μM Y27632) or DMSO control and migration was monitored. (*A*) Representative images are shown. Flow at inlet is from left to right. White dotted lines represent leading edge of monolayers. White bar = 100 μm. (*B*) Migration paths. Each red dot represents a cell. Yellow lines indicate a 15% deviation from the inlet flow direction. (*C* and *D*) For each cell, the angle between its final position and the inlet flow direction (180°) was calculated (angle deviation). (*C*) Angle deviations were divided into 20 × 18° groups and the number of cells in each group is shown. (*D*) Angle deviations, (*E*) directional persistence (DP; contour distance/Euclidean distance), and (*F*) migration velocities were calculated. Data were pooled from four independent experiments and mean values +/− SEM are shown alongside individual data points. Differences between means were compared using an unpaired *t*-test.

The effects of ROCK inhibition were also studied using EC exposed to flow on ridged slides. EC treated with Y27632 migrated further downstream from the ridge compared to controls (*Figure 4A*, compare *top* and *bottom* panels) and were capable of crossing the second ridge under ROCK inhibition (Supplementary material online, *Movie 5*). Similarly, single cell tracking analysis demonstrated that ROCK inhibition enhanced migration along the flow axis (*Figure 4B–D*), enhanced directional persistence (*Figure 4E*) and modestly heightened average migration velocity (*Figure 4F*) in EC exposed to bidirectional flow downstream from ridges. We conclude that ROCK inhibitors enhance endothelialization of ridged surfaces exposed to flow by promoting EC migration through regions of bidirectional flow into cell-free space.

**Figure 5:**
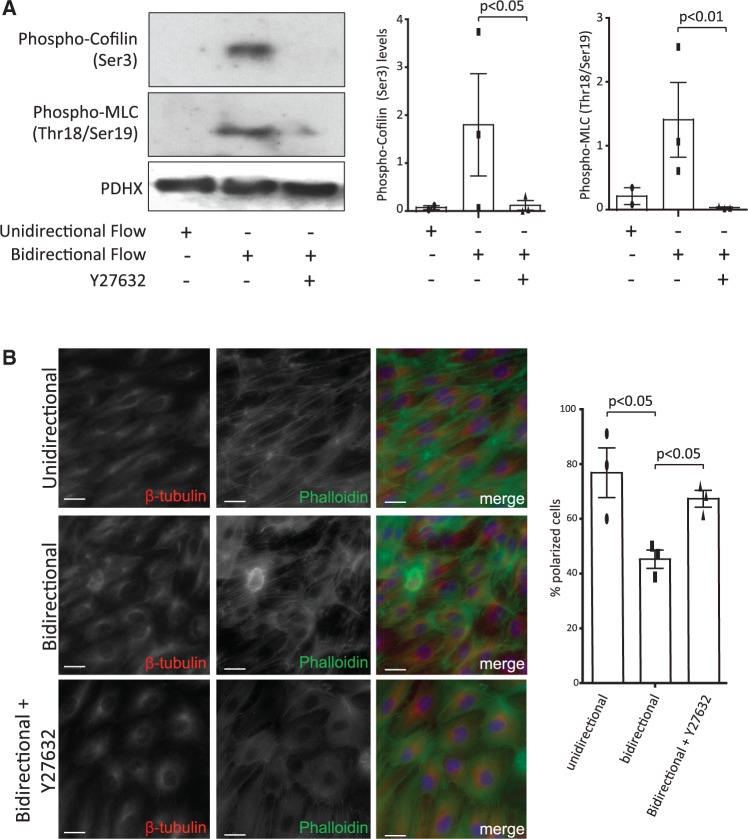
ROCK inhibition enhanced endothelial polarity under bidirectional flow by modifying actin dynamics. (*A*) HCAEC were seeded onto Ibidi slides and exposed to unidirectional or bidirectional flow in the presence or absence of a ROCK inhibitor (2 μM Y27632) for 12 h. Total cell lysates were tested by Western blotting using antibodies that detect phosphorylated forms of cofilin (Ser 3) and MLC (Thr18/Ser19) or by using anti-PDHX antibodies to assess total protein levels. Representative blots are shown. The levels of Phospho-Cofilin and Phospho-MLC were quantified by densitometric analysis. Data were pooled from three independent experiments and mean values +/− SEM are shown alongside individual data points. Differences between means were compared using a one-way ANOVA. (*B*) HUVEC were seeded onto Ibidi slides and exposed to unidirectional or bidirectional flow in the presence or absence of a ROCK inhibitor (2 μM Y27632) for 4 h. Cell polarity was assessed by immunofluorescent staining of β-tubulin (red), co-staining of actin using Phalloidin-488 (green) and co-staining of nuclei (DAPI; blue). Scale bar, 200 μm. The proportion of polarized cells (elongated morphology with the MTOC positioned upstream from the nucleus) was calculated. Data were pooled from three independent experiments and mean values +/− SEM are shown alongside individual data points. Differences between means were compared using a one-way ANOVA.

The underlying molecular mechanism was studied by assessing the effects of shear stress and ROCK inhibition on the phosphorylation of MLC and cofilin which are regulators of actin dynamics and cell polarity.^11–13^ Western blotting revealed that the levels of phosphorylated forms of MLC (activated by phosphorylation) and cofilin (inactivated) were enhanced in EC exposed to bidirectional compared to unidirectional flow (*Figure 5A*). Notably, pharmacological inhibition of ROCK reduced the levels of phosphorylated MLC and cofilin in cells exposed to bidirectional flow (*Figure 5A*), thus simultaneously reducing MLC activity while enhancing cofilin activity. These changes led us to hypothesize that ROCK inhibition may alter migratory polarity which is intimately linked to actin dynamics. This was tested by immunofluorescent staining to determine the relative positions of the nucleus, MTOC, and the actin cytoskeleton. In polarized cells, the MTOC is positioned behind the nucleus along the axis of elongation as described.^13^ It was observed that the proportion of EC that displayed polarity was reduced in cultures exposed to bidirectional compared to unidirectional flow, and that pharmacological inhibition of ROCK significantly enhanced the polarization of EC exposed to bidirectional flow (*Figure 5B*). Thus we conclude that ROCK inhibition promotes EC polarity under bidirectional flow associated with altered actin dynamics.

We also examined whether ROCK inhibitors could influence the expression of TFPI and vWF since these regulators of haemostasis are known to be expressed in sheared endothelium.^18^ Using ELISA, it was demonstrated that TFPI (Supplementary material online, *Figure S4A*) and vWF (Supplementary material online, *Figure S4B*) levels in HCAEC exposed to bidirectional flow were not significantly altered by application of the ROCK inhibitor Y27632. Thus we conclude that ROCK inhibitors promote EC migration under disturbed flow without altering TFPI or vWF expression.

### 3.4 ROCK inhibition enhanced endothelialization of a 3D *in vitro* stent model.

The ridged parallel-plate system that we developed is a highly reductionist model of EC responses to stent struts. To more closely mimic the *in vivo* situation, we determined the ability of EC to migrate over Coroflex Blue stents (65 µm strut diameter) that were deployed in PDMS tubing and subsequently exposed to flow. This 3D flow system is advantageous because it allows assessment of EC responses to complex stent geometries including flow dividers and acutely angled struts, the effects of stent materials on endothelial physiology can be monitored, and PDMS tubing is compliant thus providing a better approximation of the vascular mechanical environment compared to an inflexible 2D surface.

The influence of the flow was determined by CFD simulations using a 3D geometry generated via a PDMS cast followed by microCT imaging (Supplementary material online, *Figure S5A*). Similar to the 2D parallel-plate system, WSS values are elevated (>40 dyn/cm^2^) on the ridges of the struts with low values just downstream and upstream of the strut (Supplementary material online, *Figure S5B*). Bidirectional recirculation zones are restricted to strut orientations perpendicular to the flow direction and lead to complex streamlines lines (Supplementary material online, *Figure S5C*). For example, the flow path labelled ‘a’ in Supplementary material online, *Figure S5B* is associated with an average WSS value of 15 dyn/cm^2^ against the tubing walls. However, WSS is reduced to 0 dyn/cm^2^ just upstream of a ridge, followed by a sharp peak in WSS along the vertical plane peaking at > 50dyn/cm^2^ at the interface between the vertical and horizontal planes (plotted in Supplementary material online, *Figure S5D*). WSS remains elevated along the ridge top, then decreases sharply along the downstream vertical plane. This pattern in WSS is repeated for each strut orientation.

**Figure 6:**
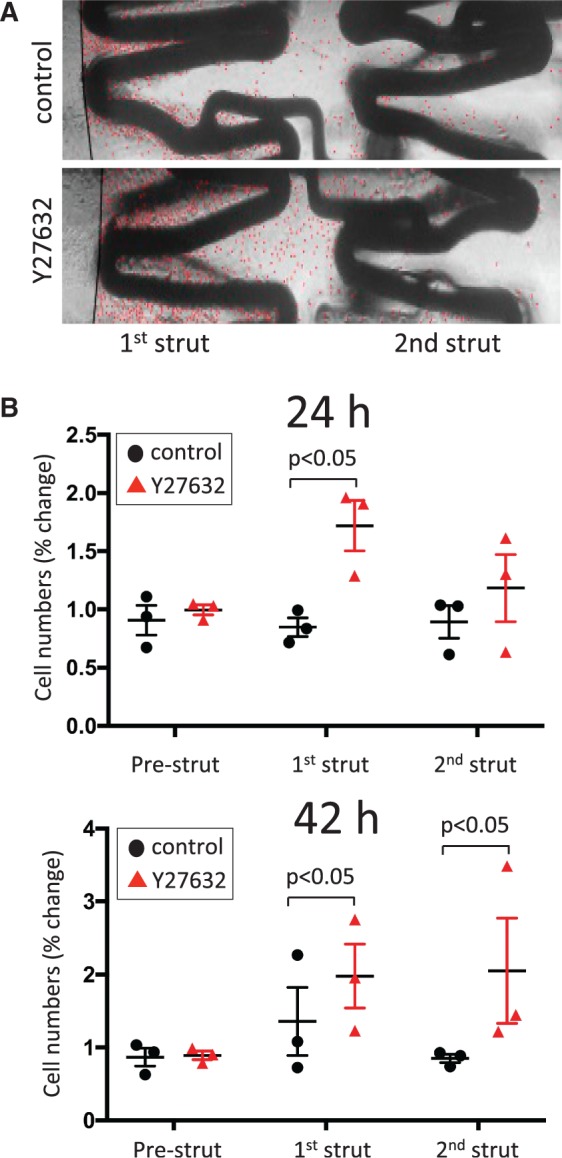
ROCK inhibition enhanced the endothelialization of a 3D *in vitro* stent model. An *in vitro* model of EC migration over stents was established by deploying Coroflex Blue stents in PDMS tubing (1.5 mm diameter). HUVEC were seeded into PDMS tubes immediately upstream from the stent. After 24 h, flow was applied (27.35 ml/min) in the presence or absence of a ROCK inhibitor (2 μM Y27632). EC coverage was assessed by brightfield microscopy at 24 h and 42 h. (*A*) Representative images are shown following 24 h exposure to flow. Individual EC are marked with a red dot to facilitate visualization. Note that EC accumulation downstream from the first strut is greater in Y27632-treated cultures compared to controls. (*B*) EC were counted at the regions located downstream from the first or second struts (and at the pre-strut region as a control). Data were pooled from three independent experiments. Mean values +/− SEM are shown alongside individual data points. Differences between means were analysed by two-way ANOVA with Sidak’s post-tests.

We hypothesized that inhibition of ROCK will increase endothelialization of stented PDMS tubes. Thus Coroflex Blue stents were deployed in fibronectin-coated PDMS tubing under sterile conditions. HUVEC were then seeded immediately upstream from the first stent strut prior to the application of flow and subsequent assessment of EC coverage by brightfield microscopy. The study revealed that the application of a ROCK inhibitor (Y27632) significantly enhanced the number of cells that accumulated downstream from the first strut at 24 h (*Figure 6A* and *B*, upper panel) and downstream from both the first and second struts at 42 h (*Figure 6B*, lower panel). We conclude that inhibition of ROCK enhanced endothelialization of stents exposed to flow under *in vitro* experimental conditions.

### 3.5 ROCK inhibition enhanced re-endothelialization of stented arteries *in vivo*

**Figure 7:**
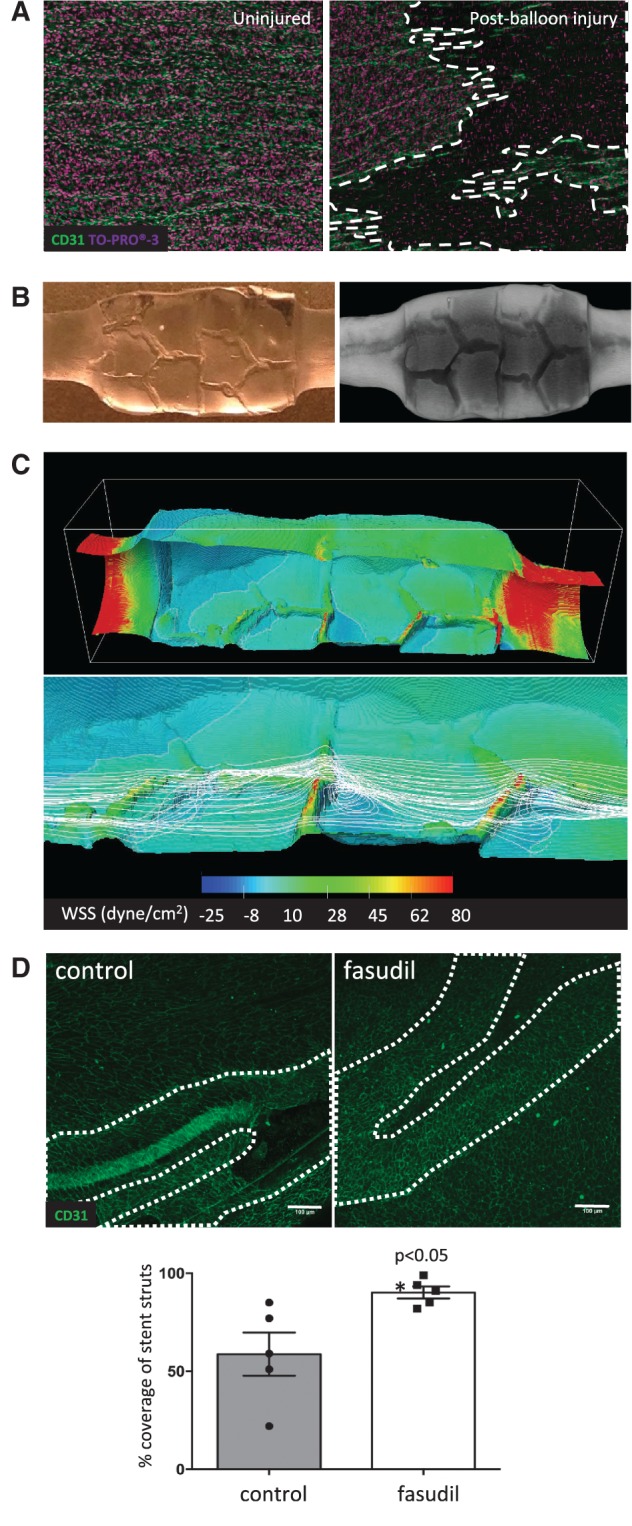
ROCK inhibition promoted re-endothelialization of stented arteries. (*A–C*) Endothelial injury in the left carotid artery was induced via repeated balloon angioplasty. A Coroflex™ stent was then deployed at the injured site. (*A*) *En face* staining of CD31 (green) and nuclei (To-Pro; purple) revealed an intact endothelial monolayer (left) in healthy arteries and loss of endothelium (dotted white lines) after balloon angioplasty (right). Data shown are representative of those obtained from *n*=5 animals in three independent experiments. (*B* and *C*) The influence on fluid dynamics was assessed. (*B*) A PDMS-based cast of the lumen of a stented carotid artery (left) and μCT (right) was performed to obtain a detailed geometry. (*C*) CFD predictions. Flow is from left to right. Upper panel shows WSS in the entire stented segment. Lower panel shows streamlines (white lines) for stent struts in relation to WSS in detail. Note high WSS at struts and low WSS corresponding to sites of recirculation downstream from struts. (*D*) The influence of fasudil on EC repair in stented arteries was determined using male Yorkshire White pigs. A portion of the carotid artery was denuded by balloon angioplasty prior to implantation of a Coroflex™ stent. Animals were treated using an osmotic pump containing either saline (vehicle group; *n* = 5 group size) or fasudil (*n* = 5 group size; 30 mg/day) for 3 days. EC coverage of the stented segment was determined by *en face* staining for CD31 followed by confocal microscopy. Representative images are shown with stent struts depicted with a broken white line (upper panels; scale bar=100μm). The percentage of EC coverage over stent struts was calculated for *n* = 5 animals per group. Data were pooled and mean values +/− SEM are presented with individual data points (lower panel). Differences between means were analysed using an unpaired *t*-test.

Next we investigated whether inhibition of ROCK can promote re-endothelialization of stented arteries *in vivo.* This study required the ability to deploy a stent over a denuded arterial segment under well-controlled experimental conditions. The porcine left carotid artery was selected for study because the geometry of this vessel is relatively uniform, allowing precise catheter-based intervention. Quantitative angiography was used to guide balloon angioplasty and subsequent stent placement over the injured segment (Supplementary material online, *Figure S6*). The influence of balloon angioplasty on endothelial coverage was assessed by *en face* staining using anti-PECAM-1 antibodies followed by confocal microscopy. It demonstrated that inflation of a balloon to approximately 1.2 times the artery diameter created vascular injury with loss of endothelial coverage and exposure of underlying smooth muscle cells, whereas intact endothelial monolayers were present in uninjured arteries (*Figure 7A*). Thus angioplasty and stent placement in the left porcine carotid artery represent a useful model to study the processes that govern EC denudation and repair.

The influence of stent deployment on WSS was determined by CFD using a geometry generated by microCT imaging of a PDMS cast (*Figure 7B*). Time-average calculations revealed a maximum velocity occurred at the centre of the lumen (0.28 m/s) with decreasing magnitude approaching the lumen surfaces. Mapping the WSS showed unidirectional highest values located upstream and downstream from the stent due to the smaller cross-sectional area at these sites (*Figure 7C*, upper panel). In the stented segment, WSS was approximately 15 dyne/cm^2^ at the inter-strut regions and the highest values (>80 dyne/cm^2^) were at the surface of the struts. Of particular note, sites downstream from the struts were associated with low WSS and recirculating flow (*Figure 7C* compare WSS (upper panel) and streamlines (lower panel) at downstream and inter-strut sites). Our observation that stent placement in carotid arteries generated recirculating flow led us to hypothesize that inhibition of ROCK may promote endothelialization *in vivo.* To investigate this, we used fasudil to inhibit ROCK *in vivo* as it is tolerated well in pigs as well as humans.^19^ Following angioplasty and stent placement in the left carotid artery, animals were treated with fasudil (30 mg/day) using an osmotic minipump or with saline as a control. LC-MS detected fasudil plasma concentrations of 93.1 ± 49.1 ng/mL in the experimental group, whereas the compound was not detected in saline-treated animals. *En face* staining using anti-PECAM-1 antibodies demonstrated that the percentage of EC coverage was 58.8 ± 11.0% in the saline-treated control group at 3 days post-stent implantation, whilst intravenous delivery of fasudil resulted in significantly increased endothelialization of the stent (90.2 ± 3.1%; *Figure 7D*). We conclude that inhibition of ROCK using fasudil significantly enhanced re-endothelialization of stented arteries *in vivo*.

## 4. Discussion

### 4.1 Strategies to enhance endothelialization of stented arteries

Endothelial loss during angioplasty and stent insertion is a major contributor to subsequent restenosis and thrombosis. Although EC repair can occur naturally, the process is gradual and may be incomplete in some situations, for example following insertion of drug-eluting stents.^1^^,^^2^^,^^6^ The corollary is that interventions to accelerate EC repair may enhance function and reduce thrombosis risk in stented arteries. Migration of local EC plays a role in re-endothelialization of injured vessels.^8^^,^^9^ For example, studies of common carotid artery allografts between transgenic mice with fluorescent endothelium (Tie2-GFP) and wild-type mice suggested that regeneration of the endothelium involved local but not bone marrow-derived EC.^9^ Similarly, a cell tracking study using mice that received either aortic or bone marrow grafts from transgenic mice with labelled EC (Tie2-LacZ) revealed that repair of the stented aorta involved both locally derived and bone marrow-derived EC, although the contribution of the latter varied between animals.^8^ Bone marrow-derived cells contribute to repair in other models including vein grafts,^20^ however their role is incompletely understood. Because of these considerations, several groups have attempted to promote re-endothelialization using stents that deliver growth factors^21^ or devices that promote capture of circulating EC precursors.^22^ However, these approaches have had either partial or negligible effects on in-stent restenosis and thrombosis,^22^ possibly because they led to incomplete endothelialization and/or because the restored EC layer was dysfunctional. Thus new strategies are required to promote endothelialization of stented arteries including novel surface chemistries^23^^,^^24^ and compounds that enhance migration of EC to the stented segment.

### 4.2 Hemodynamic regulation of stent endothelialization

**Figure 8:**
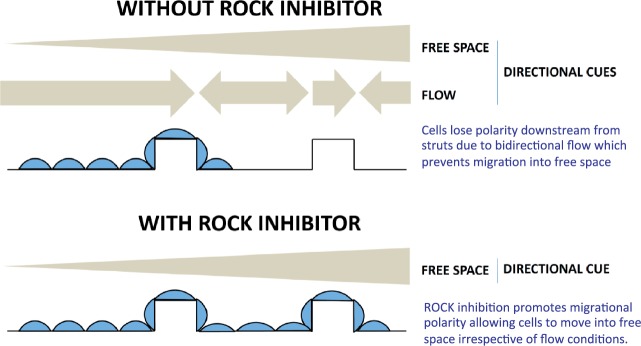
Schematic summary: ROCK inhibition promotes stent endothelialization by enhancing polarization and migration. A model is proposed. EC migrate from adjacent sites to denuded portions of stented vessels. In the absence of ROCK inhibitor, EC polarization and migration at the region upstream of stent struts are reinforced by flow which exerts a forward directional cue, whereas EC migration downstream from struts is reduced by bidirectional flow which reduces polarization. Thus EC become ‘trapped’ at the downstream bidirectional flow site. Inhibition of ROCK promotes EC polarization and therefore promotes their migration through sites of disturbed flow in stented arteries.

Here we used *in vitro* reductionist models to determine the influence of stent-induced changes in WSS on EC migration. Using a 2D parallel-plate system, it was observed that EC migrated in the direction of flow and were able to migrate over a 100 μm-high ridge that was exposed to very high WSS (> 100 dyn/cm^2^). However, EC positioned downstream from the ridge in a region exposed to flow recirculation migrated with non-uniform direction and did not move effectively into free space. The observation compliments data generated using a step-flow chamber, where EC migration distance was decreased within the flow recirculation zone.^25^ Thus EC migrate with uniform direction when they sense unidirectional mechanical cues, but migrate less efficiently when they sense bidirectional flow cues (*Figure 8*). The 2D *in vitro* model contained ridges that were 100 μm high and thus approximated the dimensions of some stent struts used clinically. With the advancement in stent fabrication, stent strut size has reduced within the last decade and recent designs are approximately 60 μm in diameter.^26^ However, bioresorbable scaffolds have considerably thicker strut sizes.^27^^,^^28^ Since thicker stent struts are associated with increased risk of complications such as thrombogenicity,^29^ the influence of stent strut geometry on local hemodynamics and EC repair remains an important consideration in stent design. Although the 2D system provided important insight into the effects of ridges on EC migration under flow, it did not capture more subtle aspects of stent geometry (e.g. rounded edges, acute strut angles) that can influence EC migration by perturbing flow. Moreover, the 2D system did not allow assessment of the effects of stent materials on EC behaviour. Because of these considerations, we developed an alternative *in vitro* model where stents were placed in fibronectin-coated tubing prior to seeding with EC and exposure to flow. This 3D system was used to validate observations made using the 2D parallel-plate chamber under with real stent geometries and materials under well-controlled experimental conditions.

### 4.3 Inhibition of ROCK promoted EC polarization and migration under disturbed flow

The 2D and 3D *in vitro* systems that we have developed represent useful platforms to test EC behaviour in response to different stent geometries, materials, or pharmacological agents. Here we used them to identify a novel pharmacological intervention, i.e. inhibition of ROCK, which promoted EC migration at sites of bidirectional flow by enhancing front-rear polarization. The ROCK family consists of two isoforms, ROCK1 and ROCK2, that act downstream from the small GTPase Ras homolog gene family member A (RhoA) to regulate cell polarity and migration by altering actin cytoskeletal organisation and dynamics.^10–12^ Of particular relevance, ROCK controls the direction of migration in EC exposed to flow.^17^^,^^18^ Using both pharmacological agents and a gene silencing approach, we examined the effects of ROCK inhibition on EC migration under flow. It was noted using either a ridged chamber or Ibidi system that ROCK inhibition enhanced EC migration under bidirectional flow by causing cells to migrate straighter (enhanced directional persistence) towards free-space. However, we also noted differences in EC behaviour between these systems. For example, inhibition of ROCK enhanced EC migration velocity by approximately 20% in ridged slides but did not in the parallel-plate system. It is likely that this relates to differences in the mechanical environment generated by these systems since EC are exquisitely sensitive to relatively small changes in shear direction or magnitude. Since the effects of ROCK inhibition on migration velocity were relatively modest and restricted to a single in vitro system and not reported by others,^17^^,^^18^ we propose that ROCK inhibition promotes endothelialization of ridged surfaces under flow predominantly by reducing changes in the direction of migrating EC.

Live cell imaging studies revealed that ROCK inhibitors suppressed EC mechanotaxis by uncoupling the direction of migration from mechanical cues, whilst retaining kenotaxis which describes the capacity of cells to move into free space (*Figure 8*). At a molecular level, the mechanism was shown to involve changes in migratory polarity and altered activity of MLC and cofilin. It was observed that EC exposed to bidirectional flow displayed reduced polarity associated with increased MLC activity compared to cells under unidirectional flow. Although MLC is required for the formation of a single protrusion in migrating cells, high MLC phosphorylation leads to the induction of multiple protrusions and stable adhesion thereby reducing polarized migration.^11^^,^^12^ We therefore propose that bidirectional flow induces excessive MLC activity which prevents the establishment of migratory polarity thus impeding EC migration into free space. Notably, ROCK inhibition counteracted the effects of bidirectional flow by reducing MLC phosphorylation, thereby enhancing polarization and migration.

MLC phosphorylation is positively regulated by MLC kinase as well as ROCK.^11^^,^^16^ However, previous reports demonstrated that migratory polarity can be enhanced by inhibition of ROCK but not by inhibition of MLC kinase.^11^^,^^16^ Similarly, we found that pharmacological inhibition of MLC kinase using ML7 did not enhance migration of HUVEC exposed to bidirectional flow (data not shown). Thus although ROCK inhibition enhanced EC migration under bidirectional flow via a mechanism that involved MLC inactivation, the inhibition of MLC activity *per se* was not sufficient to alter migration. This implies that ROCK inhibition enhances migration by targeting other proteins besides MLC. A candidate target is cofilin which is required for migratory polarity in multiple cell types.^12^^,^^30^ Moreover we observed that cofilin was inactivated via phosphorylation in response to bidirectional flow and that inhibition of ROCK normalized cofilin activity. Thus ROCK inhibitors promote EC migration through bidirectional flow towards free space by simultaneously enhancing the activity of cofilin and preventing excessive activation of MLC to enhance migratory polarization. It should be noted that complete inhibition of ROCK prevents cellular migration because ROCK is required for actinomyosin contractility.^31^^,^^32^ Consistent with this, we observed that complete silencing of ROCK1/2 using high doses of siRNA prevented EC migration (data not shown) whereas partial silencing using lower doses enhanced migration under bidirectional flow (*Figure 3*). Thus we propose that partial inhibition of ROCK enhances migratory polarity of EC under disturbed flow by altering the activity of MLC and cofilin, whilst retaining sufficient actinomyosin activity for the generation of traction forces.

While we have focused on shear forces, a recent study by Canver *et al.*^33^ revealed that collective EC migration is also tightly coupled to matrix stiffness. Stiff matrices generated high traction forces that reduced junctional integrity, whereas matrices of intermediate stiffness better supported collective cell migration because intercellular junctions remained intact. Interestingly, inhibition of ROCK promoted EC migration distance on soft substrates and increased directional persistence on both soft and stiff substrates. Thus it will be of interest in future studies to examine the functional interplay between shear stress and substrate stiffness in regulating ROCK activity and its downstream effects on EC migration. This has relevance to endothelialization of stented arteries since this intervention modifies vascular wall stiffness as well as shear stress.

### 4.4 Inhibition of ROCK enhanced stent endothelialization

In order to test the effect of ROCK inhibition on vascular repair in a stented artery *in vivo*, endothelial injury was performed via balloon angioplasty prior to stent deployment. We chose to study the carotid artery because it has a relatively straight anatomy with minimal movement throughout a cardiac cycle, thus allowing accurate positioning of balloon injury and subsequent stent deployment via angiography. It was observed using this model that systemic administration of the ROCK inhibitor fasudil significantly enhanced re-endothelialization of stented carotid arteries. This observation compliments a previous study demonstrating that systemic administration of fasudil reduced neointimal formation in stented porcine coronary arteries by promoting vascular smooth muscle cell apoptosis and reducing inflammation.^19^ Although the latter study did not report an effect of fasudil on endothelial coverage this could be because early time points were not tested. Taken together, these and our observations indicate that inhibition of ROCK may be beneficial in the context of stenting by enhancing re-endothelialization and by simultaneously reducing vascular smooth muscle cell accumulation and restenosis. It is notable that the beneficial effects of fasudil on endothelialization contrast with the deleterious effects of sirolimus- and paclitaxel-eluting stents which impede endothelial healing and have been associated with late thrombosis.^1^^,^^2^^,^^6^^,^^7^ In clinical studies, fasudil reduced the occurrence of cerebral vasospasm in patients with subarachnoid haemorrhage^34^ and had beneficial effects in patients with stable angina.^35^ Thus fasudil could be a candidate for repurposing to prevent restenosis and accelerate healing of vascular endothelium after angioplasty and stent placement. Further work should now be carried out to determine whether local delivery of fasudil or other ROCK inhibitors (via a drug-eluting stent or balloon, for example) can enhance endothelial repair and protect against restenosis in coronary arteries, and to compare its effectiveness with sirolimus- and paclitaxel-eluting stents.

## Supplementary material

Supplementary material is available at *Cardiovascular Research* online.

Supplementary Data

## References

[1] Habib A Finn AV. Endothelialization of drug eluting stents and its impact on dual anti-platelet therapy duration. Pharmacol Res 2015;93:22–27. 25533811 10.1016/j.phrs.2014.12.003 PMC4369425

[2] Lüscher TF Steffel J Eberli FR Joner M Nakazawa G Tanner FC Virmani R. Drug-eluting stent and coronary thrombosis: biological mechanisms and clinical implications. Circulation 2007;115:1051–1058. 17325255 10.1161/CIRCULATIONAHA.106.675934

[3] Chiastra C Morlacchi S Gallo D Morbiducci U Cárdenes R Larrabide I Migliavacca F. Computational fluid dynamic simulations of image-based stented coronary bifurcation models. J R Soc Interface 2013;10:20130193. 23676893 10.1098/rsif.2013.0193 PMC3673154

[4] LaDisa JF Jr Olson LE Molthen RC Hettrick DA Pratt PF Hardel MD Kersten JR Warltier DC Pagel PS. Alterations in wall shear stress predict sites of neointimal hyperplasia after stent implantation in rabbit iliac arteries. Am J Physiol Heart Circ Physiol 2005;288:H2465–H2475. 15653759 10.1152/ajpheart.01107.2004

[5] Van der Heiden K Gijsen FJ Narracott A Hsiao S Halliday I Gunn J Wentzel JJ Evans PC. The effects of stenting on shear stress: relevance to endothelial injury and repair. Cardiovasc Res 2013;99:269–275. 23592806 10.1093/cvr/cvt090

[6] McFadden EP Stabile E Regar E Cheneau E Ong AT Kinnaird T Suddath WO Weissman NJ Torguson R Kent KM Pichard AD Satler LF Waksman R Serruys PW. Late thrombosis in drug-eluting coronary stents after discontinuation of antiplatelet therapy. Lancet 2004;364:1519–1521. 15500897 10.1016/S0140-6736(04)17275-9

[7] Finn AV Joner M Nakazawa G Kolodgie F Newell J John MC Gold HK Virmani R. Pathological correlates of late drug-eluting stent thrombosis: strut coverage as a marker of endothelialization. Circulation 2007;115:2435–2441. 17438147 10.1161/CIRCULATIONAHA.107.693739

[8] Douglas G Van Kampen E Hale AB McNeill E Patel J Crabtree MJ Ali Z Hoerr RA Alp NJ Channon KM. Endothelial cell repopulation after stenting determines in-stent neointima formation: Effects of bare-metal vs. Drug-eluting stents and genetic endothelial cell modification. Eur Heart J 2013;34:3378–3388. 23008511 10.1093/eurheartj/ehs240 PMC3827553

[9] Hagensen MK Raarup MK Mortensen MB Thim T Nyengaard JR Falk E Bentzon JF. Circulating endothelial progenitor cells do not contribute to regeneration of endothelium after murine arterial injury. Cardiovasc Res 2012;93:223–231. 22012957 10.1093/cvr/cvr278

[10] Julian L Olson MF. Rho-associated coiled-coil containing kinases (ROCK): structure, regulation, and functions. Small GTPases 2014;5:e29846. 25010901 10.4161/sgtp.29846 PMC4114931

[11] Totsukawa G Wu Y Sasaki Y Hartshorne DJ Yamakita Y Yamashiro S Matsumura F. Distinct roles of MLCK and ROCK in the regulation of membrane protrusions and focal adhesion dynamics during cell migration of fibroblasts. J Cell Biol 2004;164:427–439. 14757754 10.1083/jcb.200306172 PMC2172229

[12] Gutjahr MC Rossy J Niggli V. Role of Rho, Rac, and Rho-kinase in phosphorylation of myosin light chain, development of polarity, and spontaneous migration of Walker 256 carcinosarcoma cells. Exp Cell Res 2005;308:422–438. 15950966 10.1016/j.yexcr.2005.05.001

[13] Mseka T Bamburg JR Cramer LP. ADF/cofilin family proteins control formation of oriented actin-filament bundles in the cell body to trigger fibroblast polarization. J Cell Sci 2007;120:4332–4344. 18042624 10.1242/jcs.017640

[14] He X, Luo L. Lattice boltzmann model for the incompressible navier-stokes equation. J Stat Phys 1997;88:927–944.

[15] Talon L Bauer D Gland N Youssef S Auradou H Ginzburg I. Assessment of the two relaxation time lattice-boltzmann scheme to stimulate stokes flow in porous media. Water Resource Res 2012;48:W04526.

[16] Wojciak-Stothard B Ridley AJ. Shear stress-induced endothelial cell polarization is mediated by rho and rac but not cdc42 or pi 3-kinases. J Cell Biol 2003;161:429–439. 12719476 10.1083/jcb.200210135 PMC2172912

[17] Takesono A Heasman SJ Wojciak-Stothard B Garg R Ridley AJ. Microtubules regulate migratory polarity through Rho/ROCK signaling in T cells. PLoS One 2010;5:e8774. 20098744 10.1371/journal.pone.0008774 PMC2808253

[18] Ensley AE Nerem RM Anderson DEJ Hanson SR Hinds MT. Fluid shear stress alters the hemostatic properties of endothelial outgrowth cells. Tissue Eng Part A 2012;18:127–136. 21787250 10.1089/ten.tea.2010.0290 PMC3246409

[19] Matsumoto Y Uwatoku T Oi K Abe K Hattori T Morishige K Eto Y Fukumoto Y Nakamura K Shibata Y Matsuda T Takeshita A Shimokawa H. Long-term inhibition of rho-kinase suppresses neointimal formation after stent implantation in porcine coronary arteries: Involvement of multiple mechanisms. Arterioscler Thromb Vasc Biol 2004;24:181–186. 14592842 10.1161/01.ATV.0000105053.46994.5B

[20] Xu Q Zhang Z Davison F Hu Y. Circulating progenitor cells regenerate endothelium of vein graft atherosclerosis, which is diminished in apoe-deficient mice. Circ Res 2003;93:76–86. 10.1161/01.RES.0000097864.24725.60 14512446

[21] Asahara T Bauters C Pastore C Kearney M Rossow S Bunting S Ferrara N Symes JF Isner JM. Local delivery of vascular endothelial growth factor accelerates reendothelialization and attenuates intimal hyperplasia in balloon-injured rat carotid artery. Circulation 1995;91:2793–2801. 7758186 10.1161/01.cir.91.11.2793

[22] van Beusekom HM Ertas G Sorop O Serruys PW van der Giessen WJ. The genous endothelial progenitor cell capture stent accelerates stent re-endothelialization but does not affect intimal hyperplasia in porcine coronary arteries. Catheter Cardiovasc Interv 2012;79:231–242. 21834062 10.1002/ccd.22928

[23] Potthoff E Franco D D'Alessandro V Starck C Falk V Zambelli T Vorholt JA Poulikakos D Ferrari A. Toward a rational design of surface textures promoting endothelialization. Nano Lett 2014;14:1069–1079. 24428164 10.1021/nl4047398

[24] Robotti F Franco D Bänninger L Wyler J Starck CT Falk V Poulikakos D Ferrari A. The influence of surface micro-structure on endothelialization under supraphysiological wall shear stress. Biomaterials 2014;35:8479–8486. 25017097 10.1016/j.biomaterials.2014.06.046

[25] Hsu PP Li S Li YS Usami S Ratcliffe A Wang X Chien S. Effects of flow patterns on endothelial cell migration into a zone of mechanical denudation. Biochem Biophys Res Comm 2001;285:751–759. 11453657 10.1006/bbrc.2001.5221

[26] Gassenmaier T Petri N Allmendinger T Flohr T Maintz D Voelker W Bley TA. Next generation coronary ct angiography: in vitro evaluation of 27 coronary stents. Eur Radiol 2014;24:2953–2961. 25038859 10.1007/s00330-014-3323-6

[27] Ellis SG Kereiakes DJ Metzger DC Caputo RP Rizik DG Teirstein PS Litt MR Kini A Kabour A Marx SO Popma JJ McGreevy R Zhang Z Simonton C Stone GW, ABSORB III Investigators. Everolimus-eluting bioabsorbable scaffolds for coronary artery disease. N Engl J Med 2015;373:1905–1915. 26457558 10.1056/NEJMoa1509038

[28] Gogas BD Yang B Passerini T Veneziani A Piccinelli M Esposito G Rasoul-Arzrumly E Awad M Mekonnen G Hung OY Holloway B McDaniel M Giddens D King SB III Samady H. Computational fluid dynamics applied to virtually deployed drug-eluting coronary bioresorbable scaffolds: clinical translations derived from a proof-of-concept. Glob Cardiol Sci Pract 2014;4:428–36. 10.5339/gcsp.2014.56 PMC4355516 25780796

[29] Kolandaivelu K Swaminathan R Gibson WJ Kolachalama VB Nguyen-Ehrenreich KL Giddings VL Coleman L Wong GK Edelman ER. Stent thrombogenicity early in high-risk interventional settings is driven by stent design and deployment and protected by polymer-drug coatings. Circulation 2011;123:1400–1409. 21422389 10.1161/CIRCULATIONAHA.110.003210 PMC3131199

[30] Mouneimne G DesMarais V Sidani M Scemes E Wang W Song X Eddy R Condeelis J. Spatial and temporal control of cofilin activity is required for directional sensing during chemotaxis. Curr Biol 2006;16:2193–2205. 17113383 10.1016/j.cub.2006.09.016

[31] Bryan BA Dennstedt E Mitchell DC Walshe TE Noma K Loureiro R Saint-Geniez M Campaigniac JP Liao JK D'Amore PA. RhoA/ROCK signaling is essential for multiple aspects of VEGF-mediated angiogenesis. FASEB J 2010;24:3186–3195. 20400538 10.1096/fj.09-145102 PMC2923346

[32] Yin L Morishige K Takahashi T Hashimoto K Ogata S Tsutsumi S Takata K Ohta T Kawagoe J Takahashi K Kurachi H. Fasudil inhibits vascular endothelial growth factor-induced angiogenesis in vitro and in vivo. Mol Cancer Ther 2007;6:1517–1525. 17513600 10.1158/1535-7163.MCT-06-0689

[33] Canver AC Ngo O Urbano RL Clyne AM. Endothelial directed collective migration depends on substrate stiffness via localized myosin contractility and cell-matrix interactions. J Biomech 2016;49:1369–1380. 26792289 10.1016/j.jbiomech.2015.12.037

[34] Shibuya M Suzuki Y Sugita K Saito I Sasaki T Takakura K Nagata I Kikuchi H Takemae T Hidaka H, Effect of at877 on cerebral vasospasm after aneurysmal subarachnoid hemorrhage. Results of a prospective placebo-controlled double-blind trial. J Neurosurg 1992;76:571–577. 1545249 10.3171/jns.1992.76.4.0571

[35] Vicari RM Chaitman B Keefe D Smith WB Chrysant SG Tonkon MJ Bittar N Weiss RJ Morales-Ballejo H Thadani U Fasudil Study G. Efficacy and safety of fasudil in patients with stable angina: A double-blind, placebo-controlled, phase 2 trial. J Am Coll Cardiol 2005;46:1803–1811. 16286163 10.1016/j.jacc.2005.07.047

