# Corrigendum

**DOI:** 10.1093/cvr/cvw230

**Published:** 2017-01-09

**Corrigendum to:** Endothelial repair in stented arteries is accelerated by inhibition of Rho-associated protein kinase [*Cardiovasc Res* 2016;**112**:689–701]

The authors of the above paper wish to inform readers that there was a spelling error in author, Heleen M. M. Van Beusekom's name as given. The paper has now been corrected online.

