# Supplementary material for: Endothelial repair in stented arteries is accelerated by inhibition of Rho-associated protein kinase
Source: Cardiovasc Res. 2016 Sep 26;112(3):689–701. doi: 10.1093/cvr/cvw210 (PMC5157135; doi:10.1093/cvr/cvw210)
Supplement: Supplementary Data [file supp_cvw210_cvw210.DC1.html]

Supplementary Data | Cardiovascular Research

## Supplementary Data

files

- Supplementary Data - zip file
