# Supplementary material for: Endothelial repair in stented arteries is accelerated by inhibition of Rho-associated protein kinase
Source: Cardiovasc Res. 2016 Sep 26;112(3):689–701. doi: 10.1093/cvr/cvw210 (PMC5157135; doi:10.1093/cvr/cvw210)

Supplementary Figure 1

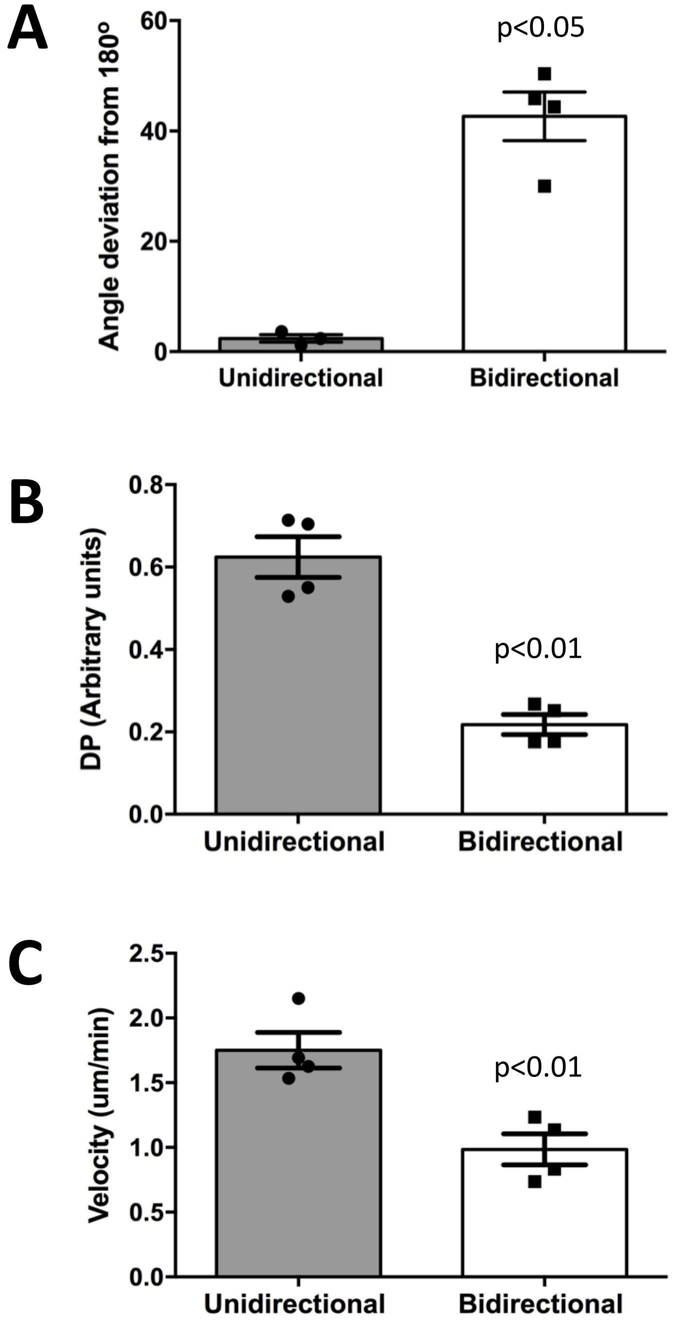

Supplementary Figure 2

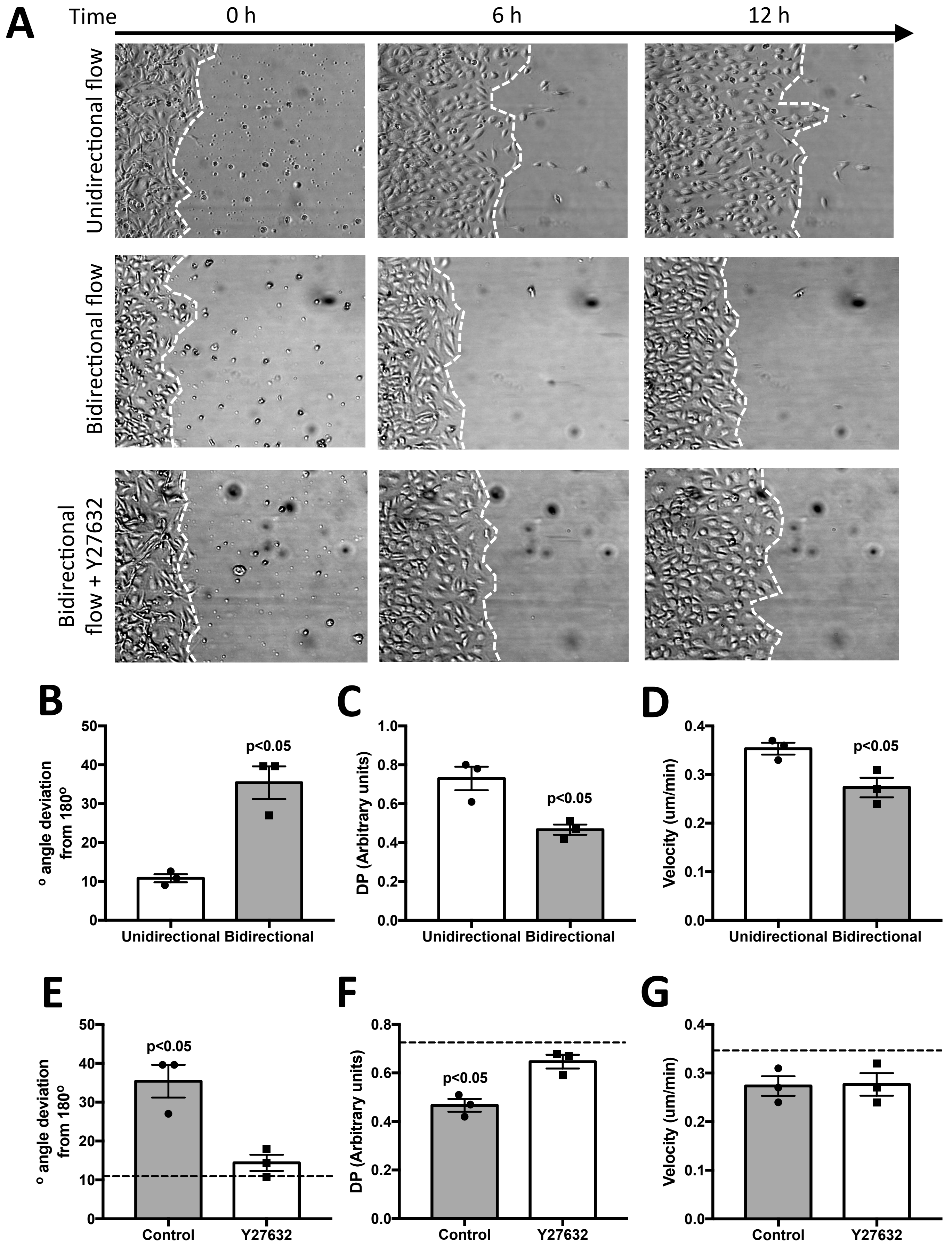

Supplementary Figure 3

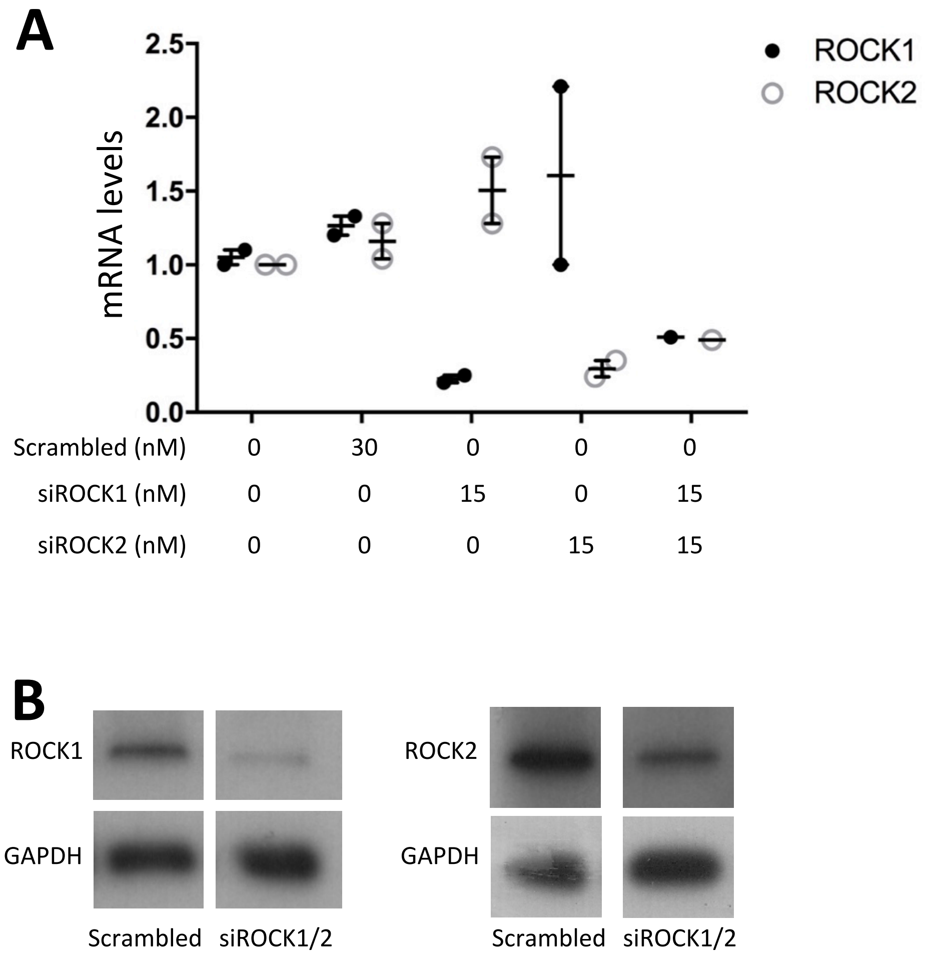

Supplementary Figure 4

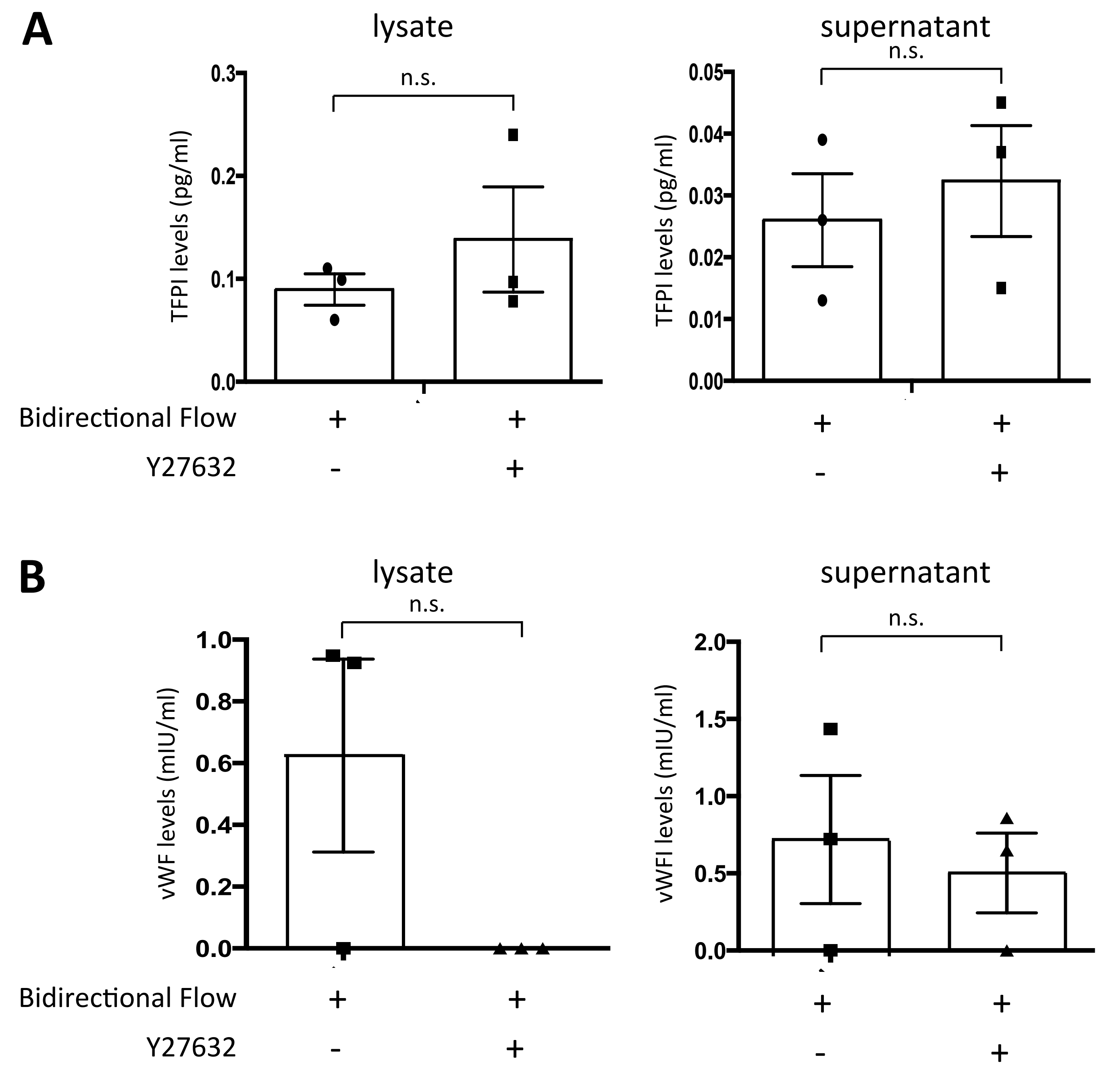

Supplementary Figure 5

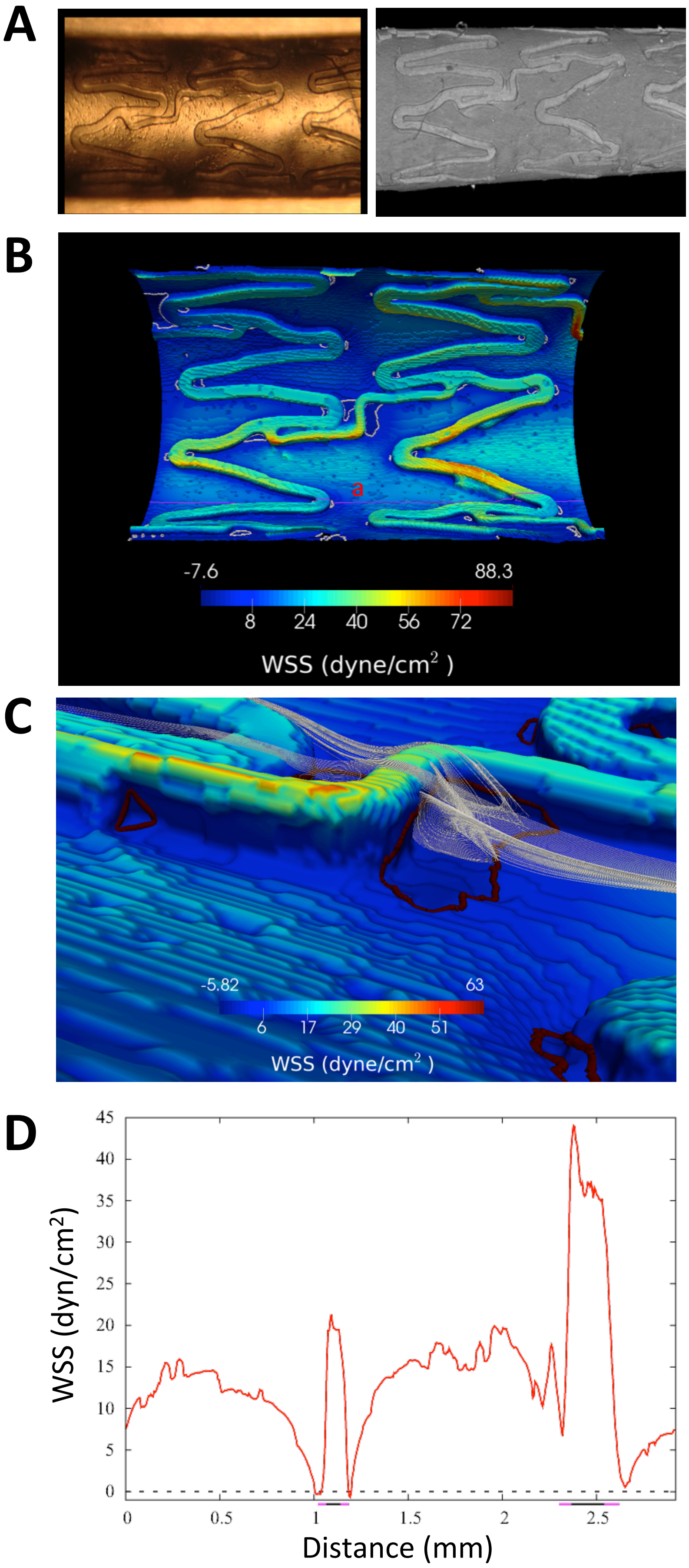

Supplementary Figure 6

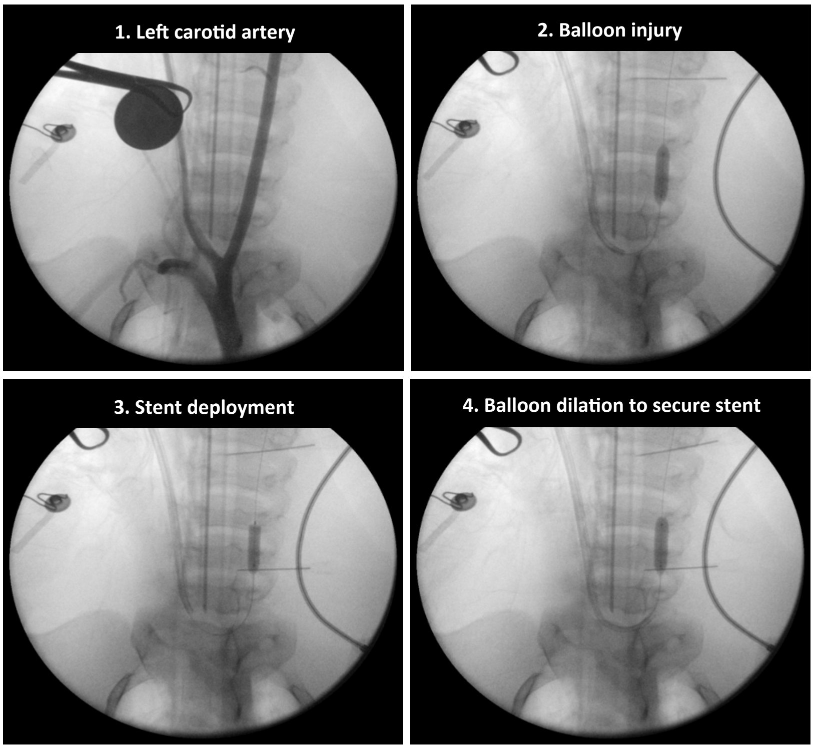

Supplement: Supplementary Data [file Suppl.zip › SUPPLEMENTARY_FIGS.pdf]
