# Supplementary material for: Endothelial repair in stented arteries is accelerated by inhibition of Rho-associated protein kinase
Source: Cardiovasc Res. 2016 Sep 26;112(3):689–701. doi: 10.1093/cvr/cvw210 (PMC5157135; doi:10.1093/cvr/cvw210)
Supplement: Supplementary Data [file Suppl.zip › SUPPLEMENTARY_MATERIALS-refs_copy.docx]

**SUPPLEMENTARY MATERIALS AND METHODS**

## **Fabrication of 2D parallel-plate flow chambers.**

## Ridged flow chambers were fabricated with polydimethylsiloxane (PDMS; Sylgard) from a KMPR 1024 (polymethylglutarimide) photoresistant (MicroChem) mould on a 3-inch silicon wafer according to a modification of a published method^1^. A high-resolution chrome mask was used to allow defined features of the mould construction. The same fabrication method was used for control (no feature) flat flow chambers. Both control and ridged PDMS chamber slides had the same inner dimension as the ibidi® µ-Slide I^0.6^ (600 µm height x 5 mm width x 50 mm length). Prior to each experiment, PDMS chamber slides were flushed with PBS, autoclaved and coated with 10 µg/ml fibronectin (Sigma-Aldrich) to allow endothelial cell (EC) attachment. Alternatively, commercial non-ridged slides with similar dimensions (600 µm height x 5 mm width x 50 mm length) were used (Ibidi® fibronectin-coated µ-Slide I^0.6,^ Ibidi GmbH).

**Computational fluid dynamics**

Fluid flow in the 2D (3D) chamber was studied using the two-dimensional (D2Q9 (D3Q19)) two-relaxation-time incompressible lattice Boltzmann (LB) method^2,3^ to solve the isothermal incompressible Navier-Stokes and Continuity equations. A TRT magic parameter^3^ of 3/16 was used and the inlet (outlet) parabolic-velocity (constant-pressure) was fixed whilst the inlet (outlet) pressure (velocity) were linearly extrapolated. LB node spacing was 10/3 *μ*m with an upstream (downstream) free region of 2 mm (2.6 mm) to avoid entrance (exit) disturbances. WSS values were obtained from quadratic extrapolations from near wall sites. The fluid was taken as Newtonian water (shear viscosity, *η* = 10^-3^ Pa.s, density, *ρ* = 10^3^ kg/m^3^) and steady flow simulations were carried out at Reynolds numbers, $Re=\frac{U^{max}H\rho}{\eta}=117$ corresponding to an open channel WSS values of 13 dyn/cm^2^ respectively (*H* = 600*μ*m, *U^max^* = maximum inlet velocity). Studies of fluid flow in stented PDMS tubes or arteries required import of cross-sectional lumen slices from μCT analysis to CFD analysis using the marker-cell method with matched voxel resolution ($Re= \bar{U}D_{h}\rho/\eta$ where $\bar{U}$= mean velocity and $D_{h}$ = mean diameter*)*. For stented PDMS tubes, WSS measurements were extrapolated as for the 2D system and taken from steady state flow *Re* = 410 with the Newtonian viscosity taken as that of culture medium (*η*= 7.3x10^-4^ Pa.s, *ρ*= 1005kg/m^3^). For stented carotid arteries, *Re* = 150 with the Newtonian viscosity taken as blood (*η*= 3.7x10^-3^ Pa.s, *ρ*= 1060kg/m^3^).

## **Particle imaging velocimetry**

The flow chamber was exposed to flowing water and fluorescently labelled polystyrene beads (FluoSpheres® carboxylate-modified microspheres 2.0 µm, blue fluorescent 365/415; Life Technologies) were introduced. Time-lapse images were captured at the rate of 20 frames per minute and a total of seven frames (15 sec interval) were merged to observe the distribution of beads over time using ImageJ®. Beads were quantified at upstream and downstream sites manually.

## **Fabrication of 3D stented model vessels.**

3D stented model vessels were fabricated with PDMS (Sylgard), moulded around a 1.5 mm metal rod. Prior to each experiment, PDMS 3D model vessels were sterilized with ethanol and coated with 10 µg/ml fibronectin (Sigma-Aldrich) to allow EC attachment. For stented 3D model vessels, Coroflex Blue stents (B. Braun Medical Ltd.) were deployed following fibronectin treatment.

**Generation of casts and μCT scanning**

Stented tubes and vessels were coated with a layer of trichloro(1H, 1H, 2H, 2H-perfluorooctyl)silane (Sigma) via evaporation deposition, to prevent the cast bonding with the vessel wall. A cast was prepared using PDMS (Sylgard 184) which was introduced into the lumen and incubated for 24 h under vacuum (to prevent accumulation of bubbles) and for a further 24 h at ambient temperature. The vessel and stent were then carefully removed. The PDMS cast was analysed using a Skyscan 1172 μCT scanner (Bruker) using the following parameters: 40 kV voltage and 149 μA current, 0.25 degree rotation step to achieve a spatial resolution of 2.96 μm. The resultant data were reconstructed and aligned using Skyscan NRecon and DataViewer software, and processed in ImageJ, to form a series of cross-sectional image slices representing the wall, stent and lumen boundary.

**Study approval**

For studies of human cells, experiments were approved by University of Sheffield Research Ethics Committee (reference SMBRER310) and all subjects gave informed consent. Studies using human cells were used in accordance to the standards set by the Declaration of Helsinki. For animal studies, all procedures were approved by the University of Sheffield ethics committee and performed in accordance with the UK Home Office Animals (Scientific Procedures) Act 1986 and in accordance with Directive 2010/63/EU of the European Parliament on the protection of animals used for scientific purposes.

## **Primary cell isolation and culture**

## Human umbilical vein EC (HUVEC) were isolated using collagenase digestion. Human coronary artery EC (HCAEC) were obtained commercially (PromoCell, Heidelberg, Germany). EC were cultured using complete growth medium (M199 medium with 20% fetal calf serum, 2 U/ml Penicillin-Streptomycin, 0.025 µg/mL Amphotericin B, 0.05 mM L-Glutamine, 10 µg/mL endothelial cell growth supplement and 0.09 µg/mL heparin) as previously described^4^.

## **ROCK inhibition in cultured EC**

Pharmacological inhibition of ROCK activity was performed using Y27632 (Calbiochem) or fasudil (5-(1,4-Diazepane-1-sulfonyl) isoquinoline; HA-1077; Calbiochem) at 2 µM and replenished every 24 h. Silencing of ROCK1 and ROCK2 was performed using small interfering RNA (siRNA; ON-TARGETplus Human ROCK1 siRNA SMARTpool and ON-TARGETplus Human ROCK2 siRNA SMARTpool), which were transfected into cells using Lipofectamine® RNAiMAX (Life Technologies) as per manufacturer’s instructions.

**Immunofluorescent staining of cultured EC.**

Cell polarity was assessed by immunofluorescent staining using antibodies against β-tubulin (Cell Signalling Technology) and Alexafluor568-conjugated secondary antibodies (Molecular Probes) to identify the microtubular organizing centre (MTOC), phalloidin-488 (Molecular Probes) to identify actin. Nuclei were identified using DAPI (Sigma). Imaging was carried out using an inverted fluorescence microscope (Olympus IX71) and image analysis was performed using Image J software (1.49p). Polarized cells were defined as those with an elongated morphology with the MTOC positioned upstream from the nucleus.

**Western blotting and ELISA.**

Total cell lysates were isolated using lysis buffer (containing 2% SDS, 10% Glycerol and 5% β-mercaptoethanol). Western blotting was carried out using specific antibodies against phosphorylated cofilin (Cell Signalling Technology), phosphorylated MLC (Cell Signalling Technology) and pyruvate dehydrogenase complex component X (PDHX; AbCam) and horse radish peroxidase-conjugated secondary antibodies (Dako). Chemiluminescent detection was carried out using ECL Prime^®^ (GE Healthcare). The protein levels of tissue factor pathway inhibitor (TFPI) and von Willebrand factor (vWF) in cell lysates or cell culture supernatants were quantified by enzyme-linked immunosorbent assay (ELISA; AbCam).

## **Live-cell imaging of EC migration under flow**

HUVEC were applied to the inlet of slides to achieve a confluent monolayer covering the proximal half (Ibidi slide) or a monolayer covering the region upstream from the first ridge (ridged slide). Flowing medium was applied using the Ibidi® pump system and chamber slides were placed on the stage of an inverted light microscope (Nikon® TE300) enclosed in a Perspex box warmed to 37°C. Time-lapse imaging (10 min per image acquisitions) was performed using the Rolera Bolt scientific CMOS camera (QImaging®) for up to 96 h. Individual cells were manually tracked using ImageJ® 1.48V software and the Chemotaxis and Migration Tool (Ibidi®) to generate migration paths and calculate the average velocity, directional persistence and position/angle relative to origin.

## **Assay of EC migration under flow using 3D stented model vessels.**

3D model vessels containing Coroflex Blue stents (B. Braun Medical Ltd.) were used. HUVEC were seeded as a confluent monolayer covering the region upstream of the first stent strut. Flowing medium was applied using the Ibidi® pump system and 3D model vessels were incubated in a 5 % CO_2_ humidified atmosphere at 37 °C. After 24 h or 42 h, EC were identified by light microscopy and the number of EC downstream from the 1^st^ or 2^nd^ struts were counted.

## **In vivo porcine stent model**

Studies were approved by the University of Sheffield Animal Welfare and Ethical Review Body. For animal studies, all procedures were approved by the University of Sheffield ethics committee and performed in accordance with the UK Home Office Animals (Scientific Procedures) Act 1986 and in accordance with Directive 2010/63/EU of the European Parliament on the protection of animals used for scientific purposes. Male Yorkshire White pigs (22-27 kg) were anaesthetised using Propofol (5 mg/kg) for induction and then maintained with inhaled isoflurane 2-3% in oxygen. Vascular access was gained via surgical cut down of the right carotid artery. Heparin (5000 U) was administered intravenously every hour. Quantitative angiography (CAAS, Pie Medical, Maastricht, Netherlands) was performed in the left carotid artery at the designated stent implantation site. To create vascular injury, a balloon (NC Euphora™, Medtronic Corp, Minneapolis, MN) was inflated 5 times for 30 sec at a balloon/artery diameter ratio of 1.2:1. A Coroflex™ stent (B Braun, Melsungen, Germany) 4.0 x 8 mm or 4.0 x 13 mm was then deployed at the designated position at 16 atm and a balloon (NC Euphora™, Medtronic Corp) was used to post-dilate the deployed stent. Surgical cut down of external jugular vein was cannulated with an Alzet® osmotic pump (DURECT Corporation), containing either saline (vehicle group; n = 5 group size) or fasudil (n = 5 group size; 30 mg/day; LC Laboratories). Aspirin (150 mg/day) and clopidogrel (75 mg/day) were given orally for 3 days following stent implantation. An angiogram was obtained at the end of the procedure to document arterial patency. The animals were killed by intravenous injection of sodium pentobarbitone (pentobarbital) after 3 days to harvest the tissue.

**Assessment of EC coverage of stent struts**

Dissected porcine carotid arteries were washed with PBS and fixed using paraformaldehyde and permeabilised (0.5% Triton-X100/5% bovine serum albumin) prior to *en face* staining using anti-PECAM-1 antibodies (LCI-4; Santa Cruz Biotech, Inc.) and Alexa Fluor® 488 goat-anti-mouse IgG antibody (Molecular Probe®). Cell nuclei were labelled using TO-PRO®-3 (Molecular Probes®). Artery segments were dissected longitudinally and Z-stack confocal microscopy was performed (Zeiss LSM 510). Raw image stacks were rendered to 3D files for outlining of stent strut. EC coverage identified with CD31^+ve^ staining was then calculated as percentage over total stent strut area (ImageJ).

## **Pharmacokinetic analysis of fasudil**

Blood samples from every animal were taken on the day of procedure and immediately prior to the sacrifice of animals to measure the plasma concentration of fasudil. The heparinised blood was centrifuged at 3000 rpm for 10 min at 4°C and plasma was stored at -80° in aliquots until analysis using the 6530 Accurate-Mass Quadrupole Time-of-Flight Liquid Chromatography/Mass Spectrometry (Agilent Technologies) as previously described^5^. Serial dilution of fasudil at known concentrations (0 – 5 µg/mL) in blank plasma was used as standards with ranitidine hydrochloride (Sigma-Aldrich) as an internal control. The concentration of fasudil plasma samples was extrapolated from standard curves using Prism6 software (Graphpad).

## **Statistical analysis**

All experiments were performed at least three times and data are presented as mean ± standard error mean. Statistical significance was determined using either Student’s *t-test* or *one-way* ANOVA followed by a *Bonferroni* multiple comparison *post hoc* test when appropriate using the Prism6 software. A *P* value of less than 0.05 was considered to be statistically significant.

**REFERENCES FOR SUPPLEMENTARY METHODS**

1. Weibel DB, Diluzio WR, Whitesides GM. Microfabrication meets microbiology. *Nat Rev Microbiol.* 2007;**5**:209-218

2. He XL, L. Lattice boltzmann model for the incompressible navier-stokes equation. *J Stat Phys.* 1997;**88**:927-944

3. Talon L, Bauer D, Gland N, Youssef S, Auradou H, Ginzburg I. Assessment of the two relaxation time lattice-boltzmann scheme to stimulate stokes flow in porous media. *Water Resource Res.* 2012;**48**:W04526

4. Partridge J, Carlsen H, Enesa K, Chaudhury H, Zakkar M, Luong L, Kinderlerer A, Johns M, Blomhoff R, Mason JC, Haskard DO, Evans PC. Laminar shear stress acts as a switch to regulate divergent functions of nf-kappab in endothelial cells. *FASEB J.* 2007;**21**:3553-3561

5. Chen H, Lin Y, Han M, Bai S, Wen S. Simultaneous quantitative analysis of fasudil and its active metabolite in human plasma by liquid chromatography electro-spray tandem mass spectrometry. *J Pharm Biomed Anal.* 2010;**52**:242-248.

**VIDEO FILES**

Supplementary Movie 1. HUVEC migration on a PDMS flat chamber slide under flow.

Supplementary Movie 2. HUVEC migration on a PDMS ridged chamber slide under flow.

Supplementary Movie 3. HUVEC migration on an Ibidi slide under unidirectional flow.

Supplementary Movie 4. HUVEC migration on an Ibidi slide under bidirectional flow.

Supplementary Movie 5. HUVEC migration on PDMS ridged chamber slide with ROCK inhibitor.
