# Supplementary material for: Endothelial repair in stented arteries is accelerated by inhibition of Rho-associated protein kinase
Source: Cardiovasc Res. 2016 Sep 26;112(3):689–701. doi: 10.1093/cvr/cvw210 (PMC5157135; doi:10.1093/cvr/cvw210)
Supplement: Supplementary Data [file Suppl.zip › Legends_for_Supplementary_Figures.docx]

**Supplementary Figure 1 Bidirectional flow modified migration behavior of HUVEC.**

HUVEC were seeded onto Ibidi slides, exposed to unidirectional or bidirectional flow and migration was monitored. For each cell, the angle between its final position and the inlet flow direction (180°) was calculated (angle deviation). **(A)** Angle deviations, **(B)** directional persistence (DP; contour distance/Euclidean distance) **(C)** and migration velocities were calculated. Data were pooled from four independent experiments and mean values +/- SEM are shown alongside individual data points. Differences between means were compared using an unpaired t-test.

**Supplementary Figure 2.** **ROCK inhibition enhanced HCAEC migration into cell-free space under bidirectional flow.**

HCAEC were seeded onto Ibidi slides and exposed to unidirectional or bidirectional flow in the presence of a ROCK inhibitor (2 μM Y27632) or vehicle-alone and migration was monitored. **(A)** Representative images are shown. Flow at inlet is from left to right. White dotted lines represent leading edge of monolayers. **(B-D)** Migration under unidirectional versus bidirectional flow was compared in control HCAEC cultures (not treated with ROCK inhibitor). **(B)** Angle deviations (the angle between the final position of each cell and the inlet flow direction), **(C)** directional persistence (DP; contour distance/Euclidean distance) and **(D)** migration velocities were calculated. **(E-G)** The effect of ROCK inhibition on HCAEC migration under bidirectional flow was assessed by comparing cells treated with Y27632 versus untreated. **(E)** Angle deviations, **(C)** DP and **(D)** migration velocities were calculated. Dotted line represents mean values from cells exposed to unidirectional flow. Data were pooled from three independent experiments and mean values +/- SEM are shown alongside individual data points. Differences between means were compared using an unpaired t-test.

**Supplementary Figure 3. Validation of ROCK1 and ROCK2 gene silencing.**

Silencing of ROCK1 or ROCK2 was performed using siRNAs. **(A)** HUVEC were treated with varying concentrations of siRNA targeting ROCK1 or ROCK2 or with non-targeting scrambled sequences. After 72 h, transcript levels of ROCK1 or ROCK2 were quantified by qRT- PCR. Data were pooled from three independent experiments and mean levels +/- SEM are shown together with individual data points. **(B)** EC were co-treated with siRNA against ROCK1 and ROCK2 (siROCK1/2) or with non-targeting scrambled sequences. The expression of ROCK1 and ROCK2 protein was determined by Western blotting using anti-GAPDH antibodies to control for total protein levels. Data shown are representative of those generated in three independent experiments.

**Supplementary Figure 4. Inhibition of ROCK did not alter expression of TFPI and vWF in EC exposed to flow.**

HCAEC were seeded onto Ibidi slides and exposed to unidirectional or bidirectional flow in the presence or absence of a ROCK inhibitor (2 μM Y27632) for 12 h. The levels of TFPI (A) or vWF (B) proteins were quantified in total cell lysates (upper panel) or cell culture supernatants (lower panel) by ELISA. Data were pooled from three independent experiments and mean values +/- SEM are shown alongside individual data points. Differences between means were compared using an unpaired t-test.

**Supplementary Figure 5. Computational fluid dynamics analysis of a 3D in vitro stent model. (A)** A PDMS-based cast of the internal dimensions of a stented PDMS tube (left) and μCT (right) was performed to obtain a detailed geometry. **(B, C)** CFD predictions. Flow is from left to right. **(B)** shows WSS in the stented segment. **(C)** shows streamlines (white lines) for stent struts in relation to WSS in detail. Note high WSS at struts and low WSS corresponding to sites of recirculation downstream from struts. **(D)** Shear stress values plotted against distance for the streamline labeled ‘a’ in panel (B).

**Supplementary Figure 6. A porcine model of endothelial injury and stent placement.**

Quantitative carotid angiography was carried out. Endothelial injury in the left carotid artery was induced via repeated balloon angioplasty (1 and 2). A Coroflex™ stent was then deployed at the injured site with guidance from anatomical and external markers (image 3). Balloon angioplasty was repeated to secure the stent (image 4).
